# Comprehensive metabolomics and lipidomics profiling uncovering neuroprotective effects of *Ginkgo biloba* L. leaf extract on Alzheimer’s disease

**DOI:** 10.3389/fphar.2022.1076960

**Published:** 2022-12-21

**Authors:** Li-Wei Liu, He-Ying Yue, Jing Zou, Meng Tang, Fan-Mei Zou, Zhuo-Lun Li, Qing-Quan Jia, Yu-Bo Li, Jian Kang, Li-Hua Zuo

**Affiliations:** ^1^ Department of Pharmacy, the First Affiliated Hospital of Zhengzhou University, Zhengzhou, Henan Province, China; ^2^ Henan Key Laboratory of Precision Clinical Pharmacy, Zhengzhou, Henan Province, China; ^3^ Henan Engineering Research Center of Clinical Mass Spectrometry for Precision Medicine, Zhengzhou, Henan Province, China; ^4^ The First Department of Orthopaedics, Zhengzhou Central Hospital Affiliated to Zhengzhou University, Zhengzhou, Henan Province, China; ^5^ Tianjin University of Traditional Chinese Medicine, Tianjin, China

**Keywords:** *Ginkgo biloba* L. leaf extract, Alzheimer’s disease, metabolomics, lipidomics, neuroprotective effects

## Abstract

**Introduction:** *Ginkgo biloba* L. leaf extract (GBLE) has been reported to be effective for alleviating cognitive and memory impairment in Alzheimer’s disease (AD). Nevertheless, the potential mechanism remains unclear. Herein, this study aimed to explore the neuroprotective effects of GBLE on AD and elaborate the underlying therapeutic mechanism.

**Methods:** Donepezil, the most widely prescribed drug for AD, was used as a positive control. An integrated metabolomics and lipidomics approach was adopted to characterize plasma metabolic phenotype of APP/PS1 double transgenic mice and describe the metabolomic and lipidomic fingerprint changes after GBLE intervention. The Morris water maze test and immunohistochemistry were applied to evaluate the efficacy of GBLE.

**Results:** As a result, administration of GBLE significantly improved the cognitive function and alleviated amyloid beta (Aβ) deposition in APP/PS1 mice, showing similar effects to donepezil. Significant alterations were observed in metabolic signatures of APP/PS1 mice compared with wild type (WT) mice by metabolomic analysis. A total of 60 markedly altered differential metabolites were identified, including 28 lipid and lipid-like molecules, 13 organic acids and derivatives, 11 organic nitrogen compounds, and 8 other compounds, indicative of significant changes in lipid metabolism of AD. Further lipidomic profiling showed that the differential expressed lipid metabolites between APP/PS1 and WT mice mainly consisted of phosphatidylcholines, lysophosphatidylcholines, triglycerides, and ceramides. Taking together all the data, the plasma metabolic signature of APP/PS1 mice was primarily characterized by disrupted sphingolipid metabolism, glycerophospholipid metabolism, glycerolipid metabolism, and amino acid metabolism. Most of the disordered metabolites were ameliorated after GBLE treatment, 19 metabolites and 24 lipids of which were significantly reversely regulated (adjusted-*p*<0.05), which were considered as potential therapeutic targets of GBLE on AD. The response of APP/PS1 mice to GBLE was similar to that of donepezil, which significantly reversed the levels of 23 disturbed metabolites and 30 lipids.

**Discussion:** Our data suggested that lipid metabolism was dramatically perturbed in the plasma of APP/PS1 mice, and GBLE might exert its neuroprotective effects by restoring lipid metabolic balance. This work provided a basis for better understanding the potential pathogenesis of AD and shed new light on the therapeutic mechanism of GBLE in the treatment of AD.

## 1 Introduction

Alzheimer’s disease (AD) is the dominant type of dementia and manifests a progressive decline in memory and cognitive function in parallel with behavioral disorders (Knopman et al., 2021). It is characterized by classical pathophysiological hallmarks of extracellular accumulating beta-amyloid (Aβ) and intraneuronal tau-laden neurofibrillary tangles (Breijyeh and Karaman, 2020). As the fifth leading cause of death in the elderly, AD has become one of the most important public health issues (GBD 2016 Dementia Collaborators, 2019). At present, there are approximately 50 million AD cases worldwide and this number is projected to triple by 2050 with the growth of geriatric population (Scheltens et al., 2021), causing enormous burden for the public health system in the world (Wong, 2020). However, there are no cures for this disease up till now. Therefore, it is urgent and significant to decode the mechanisms of AD and discover new strategies for AD therapy. Traditional Chinese medicine (TCM), with characteristics of multi-components and multi-targets, has been applied in clinical practice for thousands of years. It has attracted increasing attention for the treatment of AD, due to the comparable efficacy and fewer side effects than conventional drugs (Sun et al., 2013). Some novel natural compounds isolated from herbs have shown neuroprotective effects and are regarded as potential anti-AD drugs (Andrade et al., 2019; Suresh et al., 2022).

As one of the most universal herbal supplements, *Ginkgo biloba* L. and its different preparations have been utilized to treat neurological and cardiovascular disorders for millennia with good effectiveness on blood stasis and chest stuffiness (Tabassum et al., 2022). It is considered to possess the efficacies of promoting blood circulation to remove blood stasis and obstacles in the channels, increasing cerebral bloodflow, and protecting nerve cells (Li et al., 2018). A standard *Ginkgo biloba* L. leaf extract (GBLE), EGb761, demonstrated significant symptomatic improvement in cognitive function and behavior in patients with mild-to-moderate dementia, and was listed in local clinical guidelines in Switzerland, Germany, and some Asian countries (Savaskan et al., 2018). Promising chemical components identified in GBLE mainly include flavonoids, terpenoids, biflavonoids, and organic acids, exhibiting a variety of pharmacological activities, such as preventing oxidative stress, increasing cerebral blood flow, protecting Aβ toxicity, affecting neurotransmission and neuroplasticity (Tabassum et al., 2022). Accumulating clinical evidence has shown the therapeutic effects of GBLE on behavioral and psychological symptoms in AD patients (Nowak et al., 2021). Despite definite efficacy of GBLE, the underlying mechanism remains enigmatic, especially its effects on metabolic alterations of AD.

Evidences have suggested that metabolic dysregulation plays an important role in the pathophysiology of AD (Poddar et al., 2021). The lipid-related abnormalities were observed in the initial report of neuropathology (Alzheimer et al., 1995), and the lipid dyshomeostasis became a focus of AD research until recent decades. Both animal and clinical studies demonstrated that disrupted lipid metabolism was closely related to the pathogenesis and progression of AD (Touboul and Gaudin, 2014; Peña-Bautista et al., 2022). However, the impact of GBEL on plasma metabolome and lipidome in AD has not been investigated. Comprehensive metabolomic analysis can holistically detect the endogenous metabolic changes in biological systems, providing a powerful approach for understanding mechanism of disease and its response to TCM. Lipidomics, an important branch of metabolomics, focuses on identifying and characterizing lipid classes, subclasses and molecular species. An integration of metabolomics with lipidomics offers a global atlas of the metabolic landscape, which is becoming an emerging tool to evaluate therapeutic effects and reveal potential mechanism of TCM.

Herein, an ultra-high-performance liquid chromatography-mass spectrometry- (UHPLC-MS-) based metabolomic and lipidomic profiling analysis was performed in the plasma of wild-type and amyloid precursor protein (APP)/presenilin-1 (PS1) double-transgenic mice, a prominent mouse model of AD. It is the first exploratory investigation on the plasma metabolome and lipidome of APP/PS1 mice treated with GBLE. This study may provide a novel insight into the neuroprotective effects of GBLE and the potential mechanisms of ameliorating AD pathology, which are significant for improving clinical outcome and development of new drugs from *Ginkgo biloba* L. for AD.

## 2 Materials and methods

### 2.1 Materials and chemicals

The standardized GBLE drops (Ginaton^®^) were manufactured by Dr. Willmar Schwabe GmbH & Co KG (Karlsruhe, Germany, batch number: 0190321), containing 40 mg/ml of a standardized dry extract of Ginkgo biloba (EGb 761^®^). The ginkgo flavone glycosides account for 24% of the exact and terpenelactones for 6%. The chemical profiling of GBLE was analyzed using ultra-high performance liquid chromatography coupled with a Q Exactive hybrid quadrupole-orbitrap high resolution mass spectrometry (UHPLC-Q-Orbitrap HRMS, Thermo Fisher Scientific, San Jose, CA, United States). The chromatographic separation was performed on an ACQUITY UPLC BEH C18 column (100 mm × 2.1 mm, 1.7 μm, Waters, United States) maintained at 40°C with a flow rate of 0.20 ml/min. The mobile phase consisted of solvent A (ultra-pure water containing 0.1% formic acid) and solvent B (acetonitrile). The gradient elution was as follows: 5% B at 0–4 min, 5%–10% B at 4–15 min, 10%–20% B at 15–30 min, 20%–40% B at 30–45 min, 40%–100% B at 45–51 min, 100% B at 51–56 min, 100%–5% B at 56–56.2 min, 5% B at 56.2–60 min. The injection volume was set to 5 μl.

A total of 21 chemical constituents in GBLE were purchased from Chengdu Must Bio-technology Co., Ltd. (Sichuan, China), with the purities ≥98%. The endogenous metabolite standards were offered by Sigma-Aldrich (St Louis, MO, United States) and J&K Scientific Ltd. (Beijing, China). Rabbit anti-Aβ anti-body was purchased from Wuhan Servicebio Technology CO, LTD. (GB111197, Wuhan, China). HPLC-grade acetonitrile, methanol and isopropanol were obtained from Fisher Scientific (Fair Lawn, NJ, United States). Donepezil hydrochloride and methyl *tert*-butyl ether (MTBE) were bought from J&K Scientific Ltd. (Beijing, China). Formic acid of chromatographic grade was supplied by Aladdin Industrial Co., Ltd. (Shanghai, China). Ammonium formate was purchased from Sigma-Aldrich (St Louis, MO, United States). Deionized water was prepared by the Milli-Q water purification system (Millipore, Shanghai, China). Other chemicals and reagents were all of analytical purity.

### 2.2 Animals and experimental design

The six-month-old male APP/PS1 double transgenic mice (B6C3-Tg) and wild type (WT) littermates were purchased from Huachuang Sino Pharmaceutical Technology Co., LTD. (Jiangsu, China). After 7 days of acclimation, the APP/PS1 mice were randomly divided into APP/PS1 group (*n* = 10), APP/PS1+Donepezil group (*n* = 10), and APP/PS1+GBLE group (*n* = 10). The littermate wild-type mice were served as WT group (*n* = 10). Mice in the APP/PS1+GBLE group were given GBLE by gavage at a dose of 50 mg/kg (10 mg/ml) daily for 90 consecutive days. The dosage was determined based on the dose conversion coefficient between mouse and human in line with previous studies (Yu et al., 2021). Mice in the APP/PS1+ Donepezil group were given donepezil orally (1 mg/kg/day) for 90 days. Mice in the APP/PS1 group and WT group were intragastrically administrated with saline solution (5 ml/kg/day) for 90 days. Design of animal experiment was shown in Supplementary Figure S1. All animal studies were conducted in accordance with the National Institutes of Health Guidelines for the Care and Use of Laboratory Animals, and the experiments were approved by the Animal Ethics Committee of Zhengzhou University (ZZU-LAC20220225[13]).

### 2.3 Morris water maze test

**FIGURE 1:**
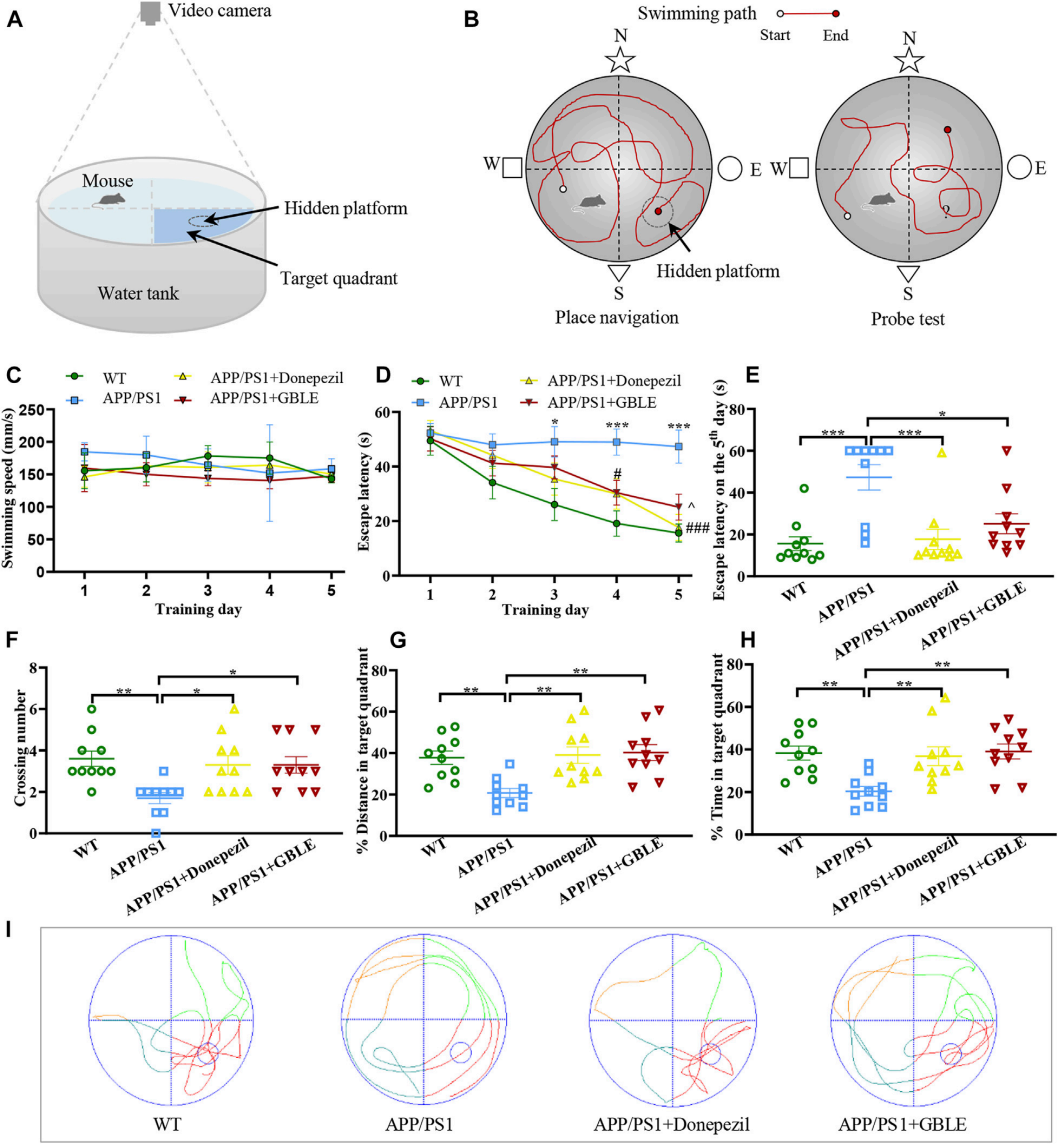
The performance of mice in Morris water maze (MWM) test. **(A)** Schematic diagram of MWM equipment. **(B)** Illustration of place navigation test and probe test in MWM. **(C)** The swimming speeds of mice in different groups during five consecutive training days. **(D)** The escape latencies of mice in different groups during five consecutive training days. ^*^: *p* < 0.05, ^***^: *p* < 0.001 for APP/PS1 group *versus* WT group; ^#^: *p* < 0.05, ^###^: *p* < 0.001 for APP/PS1+Donepezil group *versus* APP/PS1 group; ^: *p* < 0.05 for APP/PS1+GBLE group *versus* APP/PS1 group. **(E)** The escape latencies of mice in different groups on the 5th day. **(F)** The average crossing number over the platform during the probe test. **(G)** The percentage of distance in target quadrant during the probe test. **(H)** The percentage of time spent in target quadrant during the probe test. Data were presented as mean ± SEM. ^*^: *p* < 0.05, ^**^: *p* < 0.01, ^***^: *p* < 0.001. **(I)** Representative swimming paths of mice in the four groups during the probe trial.

The Morris water maze (MWM) test is one of the most common behavioral tests to assess spatial learning and memory abilities of mice. At the last week of administration in this study, the MWM test was carried out in a circular tank (diameter, 120 cm; height, 40 cm) filled with opaque water, and a hidden platform (diameter, 12 cm) was placed about 1 cm below the water surface (Figure 1A). The procedures were conducted according to previous studies with minor modifications (Yi et al., 2020).

Overall, this experiment lasted for 6 days, including place navigation test and spatial probe test (Figure 1B). In the place navigation test, mice were subjected to a spatial acquisition experiment to evaluate their spatial learning ability during a 5-day training period, and each mouse was trained to find the hidden platform twice a day with a 1-h interval. Subsequent spatial probe test was performed on the sixth day to assess spatial memory retention ability of mice, during which the mice were released into water to swim freely for 60 s after removing the submerged platform. The following data were recorded: 1) Escape latency, defined as the time for the mice to find the hidden platform area for the first time; 2) The number of crossing over the original position of platform; 3) The distance and time spent in the target quadrant for the mice. SANS video tracking system (SA201, Jiangsu, China) was applied to record and analyze the experimental data.

### 2.4 Sample collection

Two days after the MWM test, the blood samples were collected in anti-coagulation tubes by cardiac puncture and mice were sacrificed by cervical dislocation. After centrifuging at 3000 rpm at 4°C for 10 min, the supernatant plasma was transferred and stored at −80°C until metabolomic and lipidomic analysis. The brain was dissected from skull and immerged in 4% paraformaldehyde (PFA) for further immunohistochemistry assays.

### 2.5 Immunohistochemistry

The brain tissue samples were embedded in paraffin blocks, and then sectionalized at a thickness of 5 µm. The paraffin sections were deparaffinized in xylene and rehydrated in gradient ethanol. Antigen retrieval was achieved by immersing the sections in a repair box filled with citric acid buffer (PH 6.0) in a microwave oven. Subsequently, sections were sealed with 3% H_2_O_2_ to block the activity of endogenous peroxidase. The brain sections were then incubated with rabbit anti-beta-amyloid antibody diluted at 1:500 at 4 °C overnight. After washing with PBS (PH 7.4) three times for 5 min each, sections were incubated with goat anti-rabbit IgG (1:200, HRP labeled) for 50 min at room temperature. DAB color developing solution was added to the sections, and the positive showed brownish yellow. Sections were then counterstained with hematoxylin. The Images and representative photos were acquired under a microscope.

### 2.6 Untargeted metabolomic experiments

#### 2.6.1 Sample preparation

Sample preparation and data acquisition were performed according to previously published work (Liu et al., 2020). Analytes were extracted from plasma by protein precipitation with methanol for metabolomics studies. Ketoprofen and 2-chloro-l-phenylalanine were used as internal standards for the ESI^−^ and ESI^+^ modes respectively on the basis of literature (Huang et al., 2019). A 150 μL aliquot of ice-cold methanol containing 500 ng/ml ketoprofen and 50 ng/ml 2-chloro-l-phenylalanine was added to 50 μL plasma. After being vortexed for 60 s, the mixture was centrifuged at 13,000 rpm for 10 min at 4 °C. Subsequently, the supernatant was carefully transferred to a sample vial for further UHPLC-Q-Orbitrap HRMS analysis. To evaluate the robustness of analytical platform, pooled quality control (QC) sample was prepared by mixing an equal volume of each plasma sample.

#### 2.6.2 LC-MS/MS analysis

Untargeted metabolomics analysis was performed on Thermo Scientific UltiMate 3000 UHPLC system coupled to a Q-Exactive Hybrid Quadrupole-Orbitrap high resolution mass spectrometer (Thermo Scientific, San Jose, United States) equipped with a heated electrospray ionization source operating in both positive and negative ion modes as previously described (Liu et al., 2020). Chromatographic separation was achieved on a Waters ACQUITY UPLC HSS T3 column (2.1 × 100 mm, 1.8 µm), while the column oven temperature was maintained at 40 °C. The ultra-pure water containing 0.1% (v/v) formic acid (A) and acetonitrile (B) were used as mobile phase and eluted at a flow rate of 0.3 ml/min. The gradient elution was set as follows: 0–1 min, 5% B; 1–9 min, 5%–100% B; 9–12 min, 100% B; 12–12.1 min, 100%–5% B; 12.1–15 min, 5% B, and the injection volume was 5 μl. The optimized MS parameters were: ion spray voltage, 3.5 kV (+) and 2.8 kV (−); capillary temperature, 320 °C; ion source temperature, 350°C; sheath gas flow rate, 40 arb (+) and 38 arb (−); auxiliary gas flow rate, 10 arb; collision energy, 20, 40, and 60 eV. The resolution was 70,000 for full MS scan and 17,500 for dd-MS2 scan, and the scan range was performed at 80–1,200 m/z, both in positive and negative ion modes. The samples were injected in random order, and a QC sample was analyzed every five sample runs in the analytical batch. All the raw data were acquired and processed using Thermo Xcalibur 3.0 software (Thermo Scientific, San Jose, United States).

### 2.7 Lipidomic experiments

#### 2.7.1 Sample preparation

The preparation of plasma samples followed the previous publications with minor modification (Matyash et al., 2008; Chen et al., 2021). In general, aliquots of 50 μl plasma samples were pipetted into 2 ml Eppendorf tubes. A total of 300 μl pre-chilled methanol was added to each sample and vortexed for 1 min. Then, 1 ml of MTBE was added and the mixture was incubated in a shaker at room temperature for 60 min to extract the lipids. Phase separation was induced by adding 250 μl of distilled water to the mixture, followed by votexing for 30 s. After storing at room temperature for 10 min, the mixture was centrifuged at 13,000 rpm for 10 min at 4°C. Subsequently, 2 × 400 μl of the upper phase were transferred into two Eppendorf tubes respectively and dried in a vacuum centrifuge. Finally, the residues were reconstituted with 70 μl of isopropanol/acetonitrile (9:1, v/v) for LC-MS/MS analysis.

#### 2.7.2 LC-MS/MS analysis

Lipidomic analysis was also performed on Thermo Scientific UltiMate 3000 UHPLC system coupled to Q-Exactive Hybrid Quadrupole-Orbitrap high resolution mass spectrometer (Thermo Scientific, San Jose, United States). The lipid compounds separation was achieved on an ACQUITY UPLC^®^ CSH C18 column (1.7 mm × 100 mm, 1.8 µm) maitained at 40°C with a flow rate of 0.3 ml/min. The mobile phase consisted of water/acetonitrile (4:6, v/v) for solvent A and isopropanol/acetonitrile (9:1, v/v) for solvent B, with both A and B containing 10 mM ammonium formate. Analysis was carried out under gradient elution condition as follows: 0–2 min, 30%B; 2–25 min, 30%–100% B; 25–30 min, 100% B. The injection volume of each sample was 5 μl. Data was acquired using full MS/dd-MS2 approach in positive and negative ion modes, respectively. The MS parameters were the same as that of metabolomic analyses. The QC sample process identical to that of metabolomic experiment was also performed in the lipidomic analysis.

### 2.8 Data processing and statistical analysis

For the animal experiments, data was analyzed by SPSS 22.0 software (IBM Corp, Armonk, NY, United States) and GraphPad Prism 9.4.1 software (GraphPad Software Inc, San Diego, United States). Student’s t-test was used for comparisons between two groups, whereas one-way ANOVA was conducted for comparisons among groups. The data are presented as means ± standard error of the mean (SEM). A *p*-value less than 0.05 was considered statistically significant.

For the metabolomic analysis, LC-MS/MS raw data was preprocessed using Compound Discoverer (CD) 3.3 software (Thermo Fisher Scientific, San Jose, CA, United States). The workflow incorporated several defined steps, such as noise filtering, peak detection, retention time (RT) alignment, and feature annotation. The spectra were selected from raw data and then aligned with mass error of 5 ppm and RT tolerance of 0.2 min. QC samples were used for compound annotation based on fragment matching with public databases as well as additional RT against our in-house library. The mzCloud and mzVault databases were applied for compound annotation on MS/MS level with a mass tolerance of 10 ppm. ChemSpider, Human Metabolome Database (HMDB), Kyoto Encyclopedia of Genes and Genomes (KEGG), and MassList (CD internal database for endogenous metabolites) were used to annotate features based on exact mass with a mass tolerance of 5 ppm. Compound peak areas were normalized to the constant sum using embedded function before statistical analysis (Hao et al., 2018).

A data matrix including retention time (t_R_), mass-to-charge ratio (m/z) values, and peak area was generated from CD3.3 software, and the 80% rule was employed to handle missing values in the dataset. Then, the resulting three-dimensional matrix was imported into SIMICA software (version 14.1, Umetrics, Sweden) for multivariate statistical analysis, including principal component analysis (PCA) and orthogonal partial least-squares discriminant analysis (OPLS-DA). Each metabolite variable was scaled to unit variance prior to performing PCA and OPLS-DA. PCA was conducted to evaluate the overall distribution of data, evaluate reproducibility and stability of QC samples, and explore outliers. OPLS-DA was applied to screen differential features between comparable groups, and model validity was assessed by permutation test (200 permutations). Benjamini–Hochberg false discovery rate (FDR) procedure was employed for the multiple test adjustments, and adjusted-*p* values <0.05 were considered statistically significant. The differential features were selected with variable importance in the projection (VIP) value >1.0 and adjusted-*p* value <0.05. Differential metabolites were identified by searching ChemSpider, HMDB, KEGG, MassList, mzCloud, mzVault, as well as in-house-built spectral libraries based on the accurate mass, MS/MS fragments, and isotope pattern matching, and further confirmed using available reference standards.

For the lipidomic analysis, acquired MS/MS data were processed using Thermo Scientific LipidSearch 4.2 software for peak detection, lipid annotation, peak alignment, and relative quantitation (Bhawal et al., 2021). The main parameters were set as follows: precursor tolerance, 5 ppm; product tolerance, 5 ppm; intensity threshold for product ion, 5.0%. The search results from individual files were aligned using a 0.25 min tolerance window and the data merged for each annotated lipid. Subsequently, the data obtained from LipidSearch software were subjected to multivariate statistical analysis including PCA and OPLS-DA using SIMICA 14.1 software. The FDR-adjusted *p* values and fold change (FC) values were calculated according to peak areas. The differential lipids were selected based on the following criteria: VIP value >1.0 and adjusted-*p* value <0.05.

After that, the significantly altered metabolites and lipids were imported into MetaboAnalyst 5.0 software (https://www.metaboanalyst.ca/) for further analyses. Hierarchical clustering heatmaps were generated using ward’s cluster method and Euclidean distance type. Pearson correlation analyses were performed to measure the strength of associations between metabolites. The altered metabolic pathways were determined by “Pathway Analysis” module based on the *Mus musculus* KEGG pathway library, and the results from pathway enrichment analysis were combined with pathway topology analysis. Finally, a global perturbed pathway network formed with differential metabolites and lipids was depicted to reflect the overall metabolic disturbance in APP/PS1 mice and the effects of GBLE.

## 3 Results

### 3.1 Chemical composition of GBLE

The total ion chromatograms (TIC) of GBLE in positive ion mode and negative ion mode were shown in Supplementary Figure S2. A total of 81 components were identified, including 54 flavonoids and their glycosides, 13 terpenoids, 10 carboxylic acids, and 4 other compounds, 21 compounds of which were confirmed by comparison with reference standards. The chemical information of these constituents was described in Supplementary Table S1. The results showed that flavonoids and terpenoids are the main compositions in GBLE, and these components exhibited higher response in negative ion mode.

### 3.2 Effects of GBLE on the cognitive behavior and brain pathology of APP/PS1 mice

#### 3.2.1 GBLE ameliorates learning and memory impairments in APP/PS1 mice

MWM test was conducted to evaluate the neuroprotective effect of GBLE on the learning and memory deficits in APP/PS1 mice. There was no significant difference in swimming speed among all groups (Figure 1C). As shown in Figure 1D, gradually decreased escape latencies were observed over time during the five consecutive days of place navigation period for the mice in WT group, APP/PS1+Donepezil group, and APP/PS1+GBLE group. In contrast, the mice in APP/PS1 group showed no improvement in finding the hidden platform, indicating a learning impairment. Treatment with donepezil or GBLE dramatically improved their spatial learning ability after training, especially on the fifth day (Figure 1E), and GBLE exhibited a similar effect to donepezil. Evidently, the escape latency of APP/PS1 mice treated with GBLE decreased to 50.1% on the fifth day with the value of 25.14 ± 4.54 s, compared with the first day. In the probe test (without the platform), APP/PS1 mice crossed the original position of platform fewer times and spent less time in the target quadrant than WT mice, suggesting memory decline in the AD model. At the same time, treatment with GBLE significantly attenuated memory impairment of APP/PS1 mice as evidenced by increased number of crossing the original position of missing platform, elevated % distance and % time spent in the target quadrant (Figures 1F–H). In addition, Figure 1I demonstrated the typical swimming paths for different groups in the probe test on the 6th day. Together, these results indicated that GBLE treatment could noticeably ameliorate the cognitive deficits of APP/PS1 mice.

#### 3.2.2 GBLE disaggregates Aβ plaques in the brain of APP/PS1 mice

**FIGURE 2:**
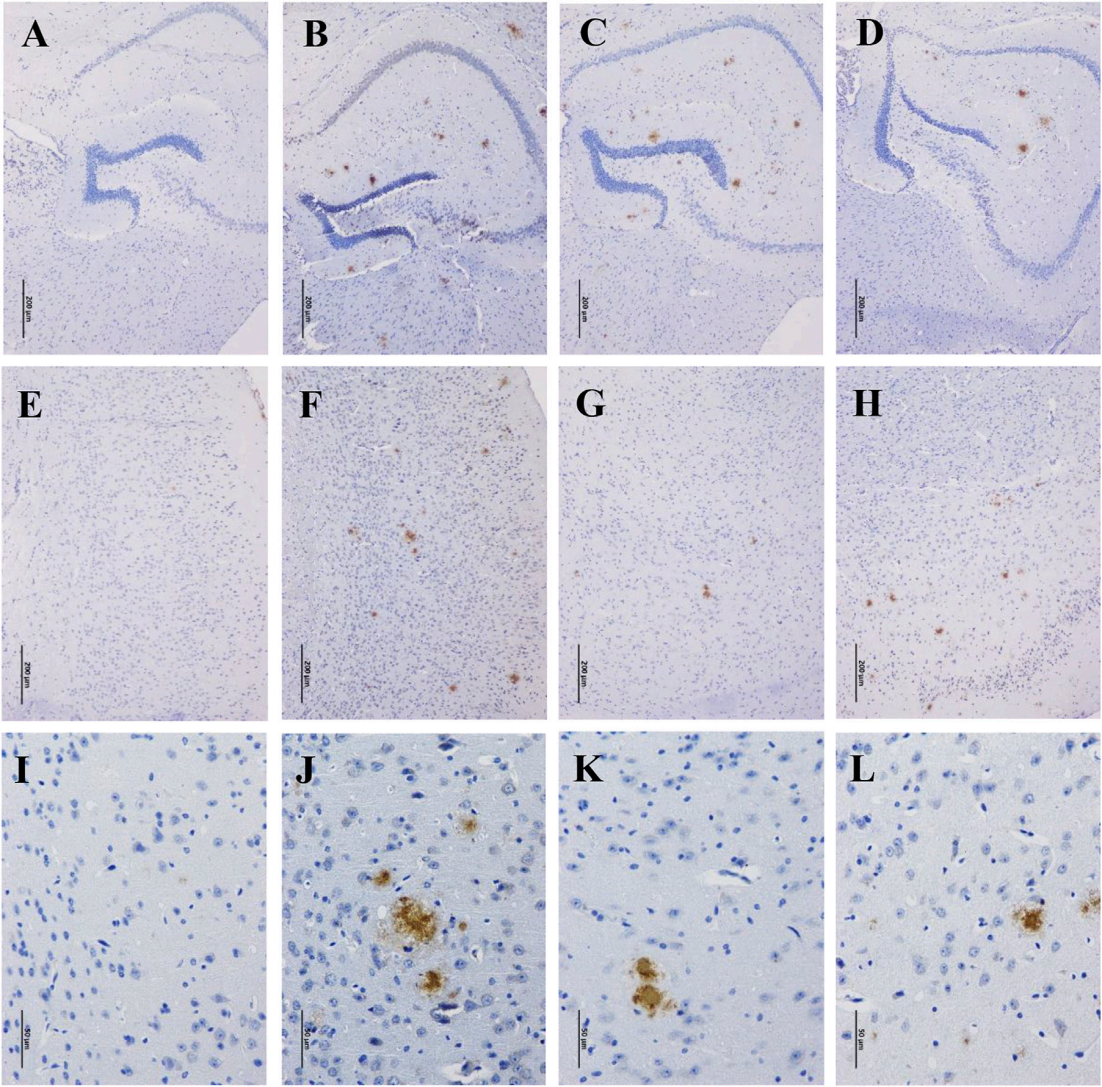
Effects of GBLE on the expression of β-amyloid in hippocampus and cortex by immunohistochemistry. **(A–D)** Immunohistochemical staining of β-amyloid plaques in hippocampus (100×) of WT, APP/PS1, APP/PS1+ Donepezil, and APP/PS1+GBLE groups, respectively. **(E–H)** Immunohistochemical staining of β-amyloid plaques in cortex (100×) for each group. **(I–L)** Immunohistochemical staining of β-amyloid plaques in cortex (400×) for each group.

Accumulation of β-amyloid aggregates in the brain is considered to be associated with cognitive impairment of AD. Therefore, the Aβ levels in the hippocampus and cortex of the mice were examined through immunohistochemistry staining. There were no Aβ deposits detected in the hippocampus and cortex of WT mice (Figures 2A,E,I), while robust Aβ plaques were clearly observed in that of APP/PS1 mice (Figures 2B,F,J). By contrast, the Aβ plaques were significantly disaggregated after 90 days of GBLE treatment (Figures 2C,G,K), exhibiting a similar result as the mice of APP/PS1+Donepezil group (Figures 2D,H,L). These results demonstrated that GBLE could alleviate Aβ accumulation and aggregate in the brain of APP/PS1 mice.

### 3.3 Global metabolic alterations in plasma samples of APP/PS1 mice and the protective effects of GBLE treatment

#### 3.3.1 Reliability Assessment of the analytical method

**FIGURE 3:**
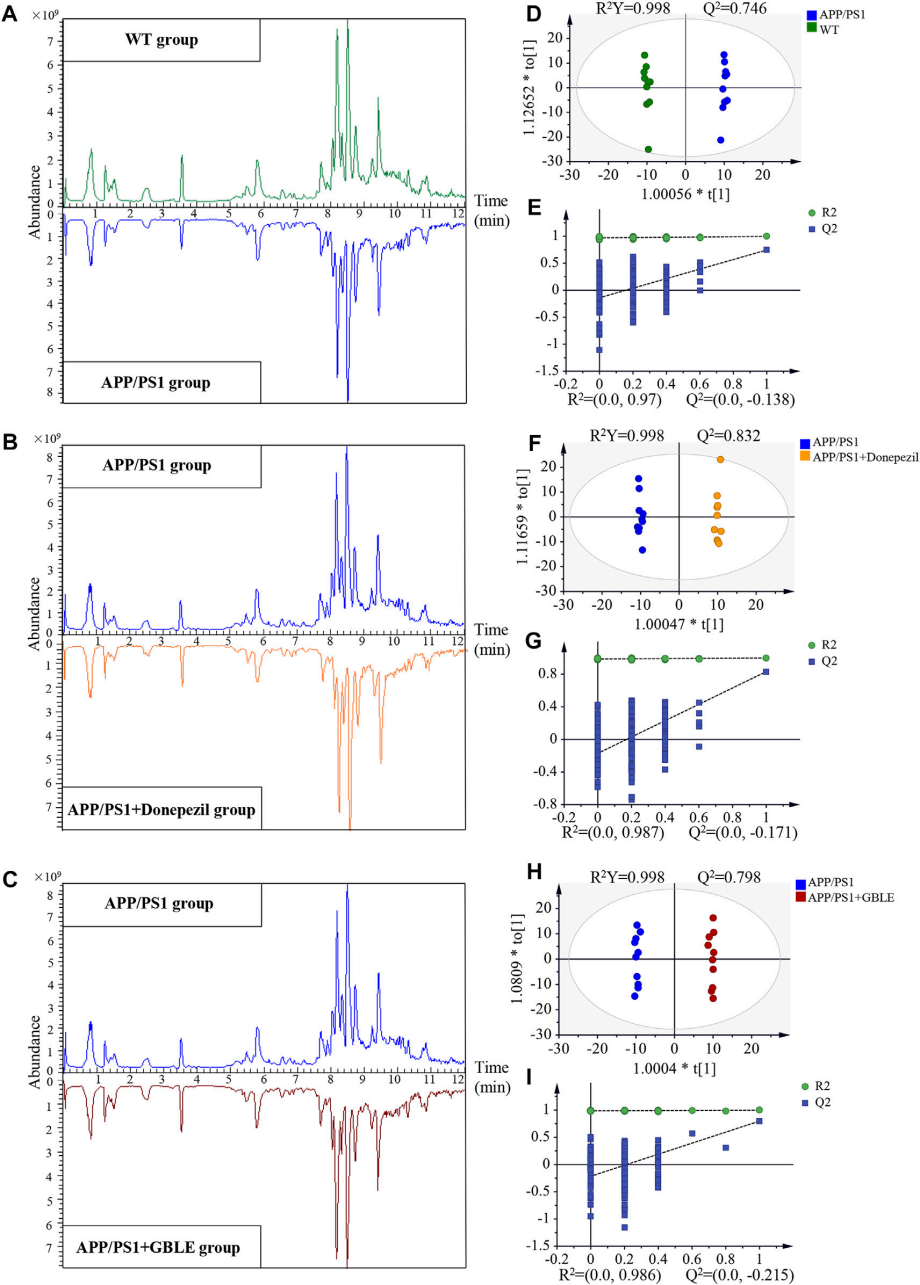
UHPLC-Q-Orbitrap HRMS-based untargeted metabolomic analysis**. (A,B,C)** Comparisons of representative total ion chromatograms for plasma samples from WT, APP/PS1, APP/PS1+Donepezil, and APP/PS1+GBLE groups in positive ion mode. **(D,E)** OPLS-DA score plots and corresponding permutation tests for APP/PS1 vs. WT in positive ion mode. **(F,G)** OPLS-DA score plots and corresponding permutation tests for APP/PS1+Donepezil vs. APP/PS1 in positive ion mode. **(H,I)** OPLS-DA score plots and corresponding permutation tests for APP/PS1+GBLE vs. APP/PS1 in positive ion mode.

Representative typical total ion chromatograms of plasma samples obtained from UHPLC-Q-Orbitrap HRMS analysis for different groups were shown in Figures 3A–C. QC samples and internal standards were applied in the current research to assess the reproducibility and stability of analytical strategy. Unsupervised PCA models for the whole dataset were generated to explore the clustering trend of all the samples. As shown in Supplementary Figures S3A,B **,** the tightly clustered QC samples in the PCA score plots both in positive and negative ion modes confirmed stability of LC-MS system and high reliability of acquired data throughout the run. In addition, over 90% (93.34% for positive ion mode and 96.82% for negative ion mode) of the ion features possessed relative standard deviation (RSD) values ≤30% across the QC samples, providing further evidence for the robustness of analytical method (Supplementary Figures S3C,D). Meantime, RSD values for the internal standards were also calculated across all the samples, and the values were 4.9% for 2-chloro-l-phenylalanine and 6.5% for ketoprofen. Supplementary Figures S3E,F represent variations of relative abundance with injection order for the ESI^+^ and ESI^−^ internal standards, respectively. The results demonstrated satisfactory stability and high reproducibility of analytical strategy in this study.

#### 3.3.2 Plasma metabolomic profiling distinguished APP/PS1 mice from WT group

**FIGURE 4:**
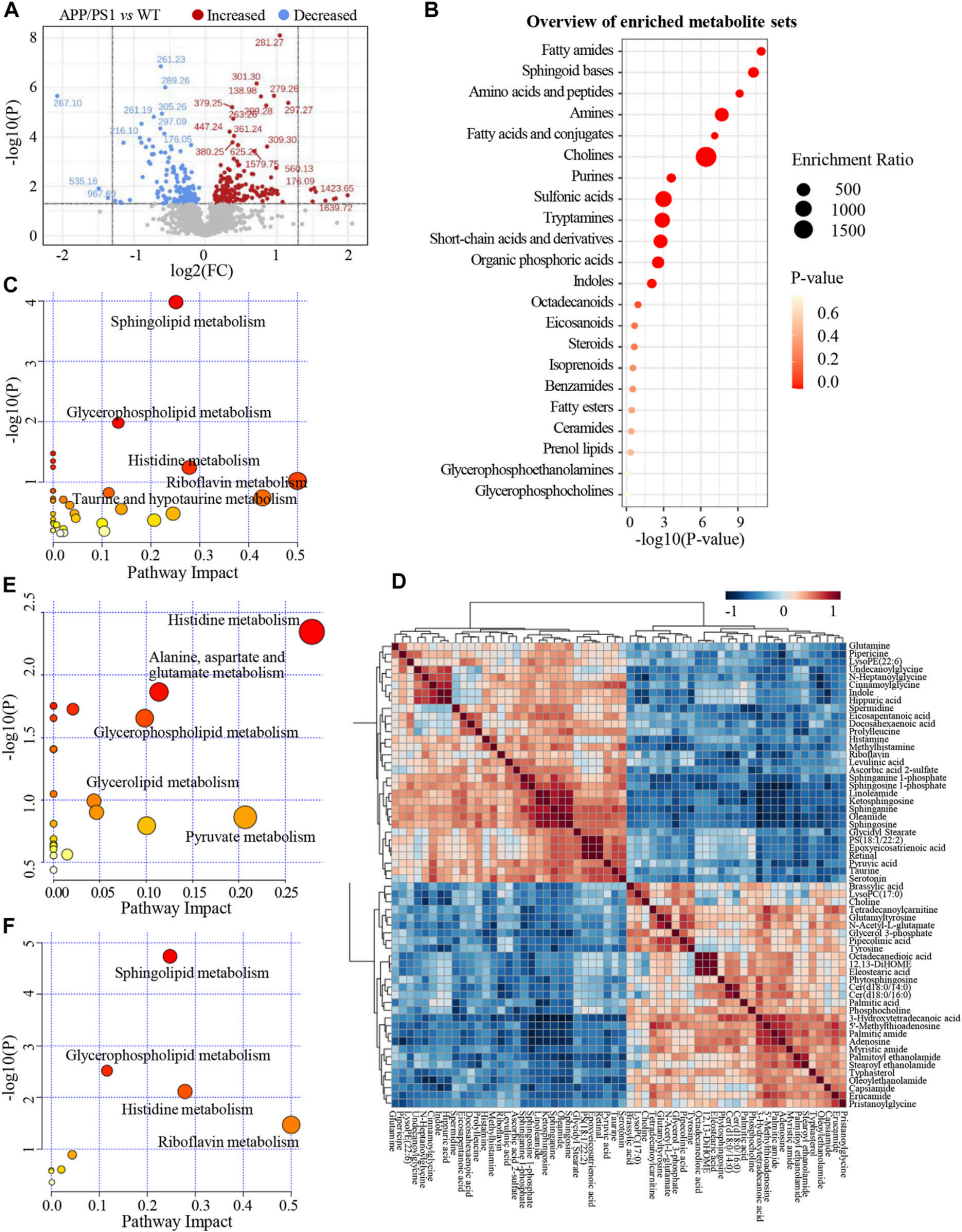
**(A)** The volcano plot of ion features for the comparison between APP/PS1 group vs. WT group. **(B)** Overview of enriched differential metabolite sets. **(C)** Metabolic pathways enrichment analysis on the basis of differential metabolites between APP/PS1 vs. WT group. **(D)** Heatmap of Pearson correlation matrix between the 60 differential metabolites. **(E)** Metabolic pathways enrichment analysis on the basis of differential metabolites between APP/PS1+GBLE vs. APP/PS1 group. **(F)** Metabolic pathways enrichment analysis on the basis of differential metabolites between APP/PS1+Donepezil vs. APP/PS1 group.

Significant discriminations in plasma metabolic phenotypes between APP/PS1 and WT groups were observed by OPLS-DA models from data produced by ESI^+^ (R^2^Y = 0.998 and Q^2^ = 0.746, Figure 3D) and ESI^−^ mode (R^2^Y = 0.998 and Q^2^ = 0.518, Supplementary Figure S4A), respectively. Furthermore, permutation tests of 200 cross-validation were performed to validate each OPLS-DA model, and the results suggested high goodness of fit and good predictive capability of the constructed models (Figure 3E, Supplementary Figure S4B). After combining positive and negative data, 305 ion features that significantly contributed to the metabolic distinction between the two groups were screened. The volcano plot graphically depicts −log10 (*p*-value) *versus* log2 (FC) for ion features between the comparison (Figure 4A).

**FIGURE 5:**
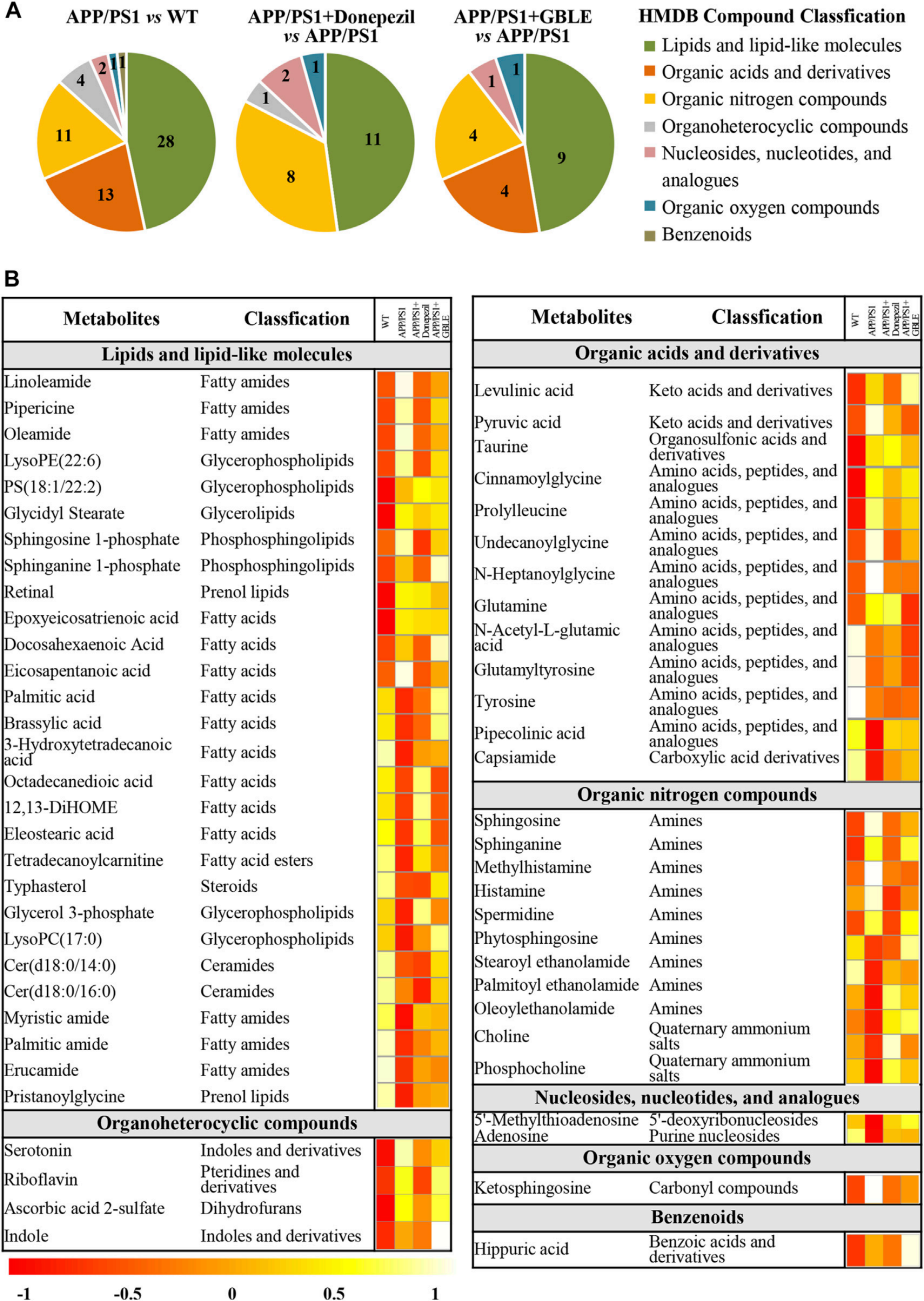
Classification and heatmap analysis of differential metabolites. **(A)** Pie charts showing the HMDB compound classification of disorded metabolites in APP/PS1 and regulated metabolites by Donepezil and GBLE, respectively. **(B)** Heat map showing the average levels of metabolites in each group. The colors from red to white in the heatmap indicate higher levels of metabolites.

As shown in Table 1, a total of 60 differential metabolites (43 metabolites detected in positive ion mode and 17 metabolites in negative mode) were identified between APP/PS1 and WT groups according to accurate *m/z* and MS/MS fragments, and 11 metabolites of which were confirmed by reference standards. Wherein the levels of 31 metabolites obviously increased in APP/PS1 mice compared with WT group, mainly including oleamide, sphinganine, linoleamide, ketosphingosine, sphingosine, and sphingosine 1-phosphate, while 29 metabolites such as 3-hydroxytetradecanoic acid, 5′-methylthioadenosine, adenosine, palmitic amide, and Cer(d18:0/14:0) exhibited significant down-regulated trends. Metabolite set enrichment analysis on the basis of a library containing 464 main chemical class metabolite sets showed that fatty amides, sphingoid bases, amino acids and peptides, amines, and fatty acids and conjugates were notably affected in APP/PS1 group (Figure 4B). The differential metabolites were classified based on HMDB database. As illustrated in Figure 5A, different colors of each pie represent different HMDB classification. The results demonstrated that these metabolites consisted of 28 lipid and lipid-like molecules, 13 organic acids and derivatives, 11 organic nitrogen compounds, and 8 other compounds, indicative of significant changes in lipid metabolism of AD.

**TABLE 1:** Differential metabolites between the comparison of APP/PS1 and WT groups and the change trends in response to GBLE treatment.

No	Differential metabolites	m/z	Rt (min)	Formula	Ion mode	APP/PS1 vs*.* WT			APP/PS1+Donepezil vs*.* APP/PS1		APP/PS1+GBLE vs*.* APP/PS1	
						VIP	Adjusted-*p* value	FC	Adjusted-*p* value	FC	Adjusted-*p* value	FC
1	Spermidine	146.16513	0.67	C_7_H_19_N_3_	P	1.39	4.26E-02	1.20 ↑^a^	9.69E-02	0.83 ↓	9.32E-01	0.98 ↓
2	Methylhistamine	126.10269	0.68	C_6_H_11_N_3_	P	1.80	1.21E-02	1.59 ↑^a^	4.47E-02	0.66 ↓^a^	2.44E-02	0.63 ↓^a^
3	Histamine^b^	112.08716	0.68	C_5_H_9_N_3_	P	1.34	4.10E-02	1.35 ↑^a^	1.63E-03	0.56 ↓^c^	4.50E-02	0.71 ↓^a^
4	Pipecolinic acid^b^	130.08627	0.71	C_6_H_11_NO_2_	P	1.39	4.26E-02	0.80 ↓^a^	1.31E-01	1.20 ↑	2.52E-02	1.19 ↑^a^
5	Choline^b^	104.10720	0.82	C_5_H_13_NO	P	1.33	4.28E-02	0.91 ↓^a^	1.14E-02	1.23 ↑^a^	1.54E-01	1.08 ↑
6	Glutamine^b^	147.07627	0.85	C_5_H_10_N_2_O_3_	P	1.36	4.28E-02	1.12 ↑^a^	7.38E-01	1.02 ↑	2.58E-02	0.87 ↓^a^
7	Taurine^b^	124.00612	0.87	C_2_H_7_NO_3_S	N	1.97	1.78E-02	1.29 ↑^a^	7.58E-01	1.03 ↑	7.63E-01	0.96 ↓
8	Prolylleucine	229.15440	0.88	C_11_H_20_N_2_O_3_	P	1.54	2.94E-02	1.31 ↑^a^	3.86E-01	0.88 ↓	6.71E-01	0.93 ↓
9	Ascorbic acid 2-sulfate	254.98130	0.89	C_6_H_8_O_9_S	N	1.64	3.69E-02	1.26 ↑^a^	4.95E-01	0.91 ↓	9.32E-01	1.02 ↑
10	Pyruvic acid	87.00742	0.95	C_3_H_4_O_3_	N	1.86	2.81E-02	1.57 ↑^a^	1.97E-01	0.78 ↓	3.44E-02	0.65 ↓^a^
11	Glycerol 3-phosphate	171.00533	0.96	C_3_H_9_O_6_P	N	2.10	1.22E-02	0.69 ↓^a^	2.45E-03	1.68 ↑^c^	4.50E-02	1.27 ↑^a^
12	Adenosine	268.10368	1.33	C_10_H_13_N_5_O_4_	P	2.48	2.67E-05	0.24 ↓^c^	4.32E-03	3.09 ↑^c^	9.84E-02	3.00 ↑
13	N-Acetyl-l-glutamate	188.05560	1.34	C_7_H_11_NO_5_	N	1.79	2.99E-02	0.63 ↓^a^	5.91E-01	1.11 ↑	3.10E-01	0.82 ↓
14	Tyrosine^b^	180.06571	1.36	C_9_H_11_NO_3_	N	1.62	3.88E-02	0.74 ↓^a^	7.66E-01	0.95 ↓	8.78E-01	0.97 ↓
15	Serotonin^b^	177.10216	1.92	C_10_H_12_N_2_O	P	1.57	2.81E-02	2.88 ↑^a^	2.62E-01	0.63 ↓	5.76E-01	0.77 ↓
16	Levulinic acid	115.03884	2.50	C_5_H_8_O_3_	N	1.47	4.49E-02	1.24 ↑^a^	3.54E-01	0.87 ↓	4.43E-01	1.11 ↑
17	Glutamyltyrosine	311.12348	2.96	C_14_H_18_N_2_O_6_	P	1.46	3.69E-02	0.63 ↓^a^	6.04E-01	1.14 ↑	6.84E-01	0.92 ↓
18	5′-Methylthioadenosine	298.09662	3.50	C_11_H_15_N_5_O_3_S	P	2.31	3.06E-04	0.65 ↓^c^	8.32E-05	1.58 ↑^c^	6.85E-03	1.74 ↑^c^
19	Riboflavin^b^	377.14523	4.10	C_17_H_20_N_4_O_6_	P	1.37	3.87E-02	1.16 ↑^a^	4.12E-02	0.88 ↓^a^	8.78E-01	1.02 ↑
20	Hippuric acid^b^	180.06544	4.19	C_9_H_9_NO_3_	P	1.42	3.69E-02	1.48 ↑^a^	3.21E-01	0.85 ↓	9.84E-02	1.44 ↑
21	Indole	118.06525	4.20	C_8_H_7_N	P	1.40	3.74E-02	1.58 ↑^a^	4.70E-01	0.86 ↓	7.87E-02	1.50 ↑
22	N-Heptanoylglycine	186.11273	4.44	C_9_H_17_NO_3_	N	2.02	1.45E-02	1.19 ↑^a^	7.09E-02	0.86 ↓	4.96E-02	0.86 ↓^a^
23	Cinnamoylglycine	204.06582	5.15	C_11_H_11_NO_3_	N	1.68	3.50E-02	1.78 ↑^a^	6.62E-01	0.87 ↓	9.32E-01	0.97 ↓
24	3-Hydroxytetradecanoic acid	262.23719	5.18	C_14_H_28_O_3_	P	2.61	4.20E-06	0.65 ↓^c^	3.63E-02	1.24 ↑^a^	1.90E-02	1.26 ↑^a^
25	Undecanoylglycine	266.17200	6.18	C_13_H_25_NO_3_	P	1.60	2.81E-02	1.29 ↑^a^	7.39E-02	0.78 ↓	4.04E-01	0.86 ↓
26	Palmitic acid	274.27325	6.56	C_16_H_32_O_2_	P	1.64	2.81E-02	0.83 ↓^a^	5.38E-01	1.08 ↑	2.58E-02	1.29 ↑^a^
27	Phytosphingosine	318.29944	6.60	C_18_H_39_NO_3_	P	1.42	3.82E-02	0.85 ↓^a^	7.55E-01	1.03 ↑	7.29E-02	1.26 ↑
28	Brassylic acid	243.15975	6.96	C_13_H_24_O_4_	N	1.77	3.26E-02	0.85 ↓^a^	5.65E-01	1.05 ↑	6.85E-03	1.24 ↑^c^
29	Linoleamide	280.26292	7.02	C_18_H_33_NO	P	2.51	2.67E-05	1.95 ↑^c^	8.32E-05	0.54 ↓^c^	1.90E-02	0.66 ↓^a^
30	Ketosphingosine	298.27348	7.02	C_18_H_35_NO_2_	P	2.47	4.21E-05	2.24 ↑^c^	6.01E-04	0.53 ↓^c^	1.90E-02	0.61 ↓^a^
31	Oleamide	282.27854	7.42	C_18_H_37_NO	P	2.70	4.70E-07	2.06 ↑^c^	2.57E-05	0.55 ↓^c^	7.87E-02	0.70 ↓
32	Sphingosine	300.28910	7.42	C_18_H_37_NO_2_	P	2.45	4.62E-05	1.81 ↑^c^	1.59E-03	0.62 ↓^c^	1.50E-01	0.74 ↓
33	Sphinganine	302.30458	7.57	C_18_H_39_NO_2_	P	2.53	1.39E-05	1.65 ↑^c^	1.59E-03	0.69 ↓^c^	9.39E-01	1.01 ↑
34	Sphingosine 1-phosphate	378.24130	7.63	C_18_H_38_NO_5_P	N	2.80	4.74E-05	1.30 ↑^c^	5.69E-04	0.72 ↓^c^	2.38E-01	0.89 ↓
35	Eleostearic acid	279.23118	7.77	C_18_H_30_O_2_	P	1.50	3.26E-02	0.63 ↓^a^	7.09E-02	1.67 ↑	7.63E-01	1.10 ↑
36	Octadecanedioic acid	337.23409	7.77	C_18_H_34_O_4_	P	1.43	3.69E-02	0.68 ↓^a^	7.09E-02	1.62 ↑	9.36E-01	1.02 ↑
37	12,13-DiHOME	313.23823	7.78	C_18_H_34_O_4_	N	1.54	4.28E-02	0.69 ↓^a^	1.06E-01	1.66 ↑	8.63E-01	1.07 ↑
38	Sphinganine 1-phosphate	382.27086	7.81	C_18_H_40_NO_5_P	P	1.91	6.74E-03	1.39 ↑^c^	3.63E-02	0.78 ↓^a^	3.93E-01	1.28 ↑
39	LysoPE (22:6)	526.29159	8.17	C_27_H_44_NO_7_P	P	1.69	1.78E-02	1.23 ↑^a^	3.63E-02	0.82 ↓^a^	4.47E-01	0.93 ↓
40	Docosahexaenoic acid^b^	327.23255	8.20	C_22_H_32_O_2_	N	2.06	1.45E-02	1.18 ↑^a^	9.69E-02	0.87 ↓	2.38E-01	1.13 ↑
41	Phosphocholine^b^	184.07265	8.56	C_5_H_14_NO_4_P	P	1.45	3.82E-02	0.68 ↓^a^	3.63E-02	1.64 ↑^a^	1.36E-01	1.46 ↑
42	Tetradecanoylcarnitine	372.31015	8.91	C_21_H_41_NO_4_	P	1.49	3.58E-02	0.85 ↓^a^	3.63E-02	1.13 ↑^a^	5.68E-01	1.06 ↑
43	Retinal	285.22070	8.91	C_20_H_28_O	P	1.53	3.26E-02	1.99 ↑^a^	9.58E-01	0.98 ↓	8.63E-01	0.89 ↓
44	PS(18:1/22:2)	840.57575	8.92	C_46_H_84_NO_10_P	N	1.80	3.23E-02	1.74 ↑^a^	5.91E-01	1.15 ↑	8.63E-01	1.10 ↑
45	Epoxyeicosatrienoic acid	319.22738	8.93	C_20_H_32_O_3_	N	1.75	3.29E-02	2.02 ↑^a^	9.12E-01	0.97 ↓	9.32E-01	0.96 ↓
46	LysoPC(17:0)	510.35456	8.98	C_25_H_52_NO_7_P	P	1.33	4.51E-02	0.82 ↓^a^	2.36E-01	1.15 ↑	1.90E-02	1.33 ↑^a^
47	Glycidyl Stearate	341.30430	9.24	C_21_H_40_O_3_	P	1.41	3.69E-02	1.21 ↑^a^	7.47E-01	0.97 ↓	9.32E-01	0.99 ↓
48	Cer(d18:0/14:0)	512.50289	9.68	C_32_H_65_NO_3_	P	1.84	9.36E-03	0.69 ↓^c^	7.69E-01	0.96 ↓	8.05E-02	1.29 ↑
49	Pipericine	336.32532	9.78	C_22_H_41_NO	P	1.78	1.45E-02	1.89 ↑^a^	3.63E-02	0.56 ↓^a^	3.93E-01	0.81 ↓
50	Eicosapentanoic acid	301.21712	9.85	C_20_H_30_O_2_	N	2.31	5.38E-03	1.36 ↑^c^	4.32E-03	0.72 ↓^c^	4.50E-02	0.82 ↓^a^
51	Palmitoyl ethanolamide	300.28907	9.86	C_18_H_37_NO_2_	P	1.78	1.45E-02	0.82 ↓^a^	3.63E-02	1.37 ↑^a^	1.90E-02	1.25 ↑^a^
52	Oleoyl ethanolamide	326.30463	10.05	C_20_H_39_NO_2_	P	1.87	1.10E-02	0.82 ↓^a^	1.89E-02	1.45 ↑^a^	3.88E-04	1.55 ↑^c^
53	Cer(d18:0/16:0)	540.53402	10.06	C_34_H_69_NO_3_	P	1.37	4.10E-02	0.77 ↓^a^	1.93E-01	0.83 ↓	4.43E-01	1.12 ↑
54	Pristanoylglycine	356.31512	10.20	C_21_H_41_NO_3_	P	1.53	3.26E-02	0.83 ↓^a^	5.91E-01	1.09 ↑	2.38E-01	1.10 ↑
55	Myristic amide	228.23177	10.42	C_14_H_29_NO	P	1.61	2.81E-02	0.66 ↓^a^	3.63E-02	1.36 ↑^a^	3.44E-02	1.33 ↑^a^
56	Capsiamide	270.27852	10.46	C_17_H_35_NO	P	1.60	2.81E-02	0.84 ↓^a^	5.65E-01	1.09 ↑	2.91E-01	1.11 ↑
57	Stearoyl ethanolamide	328.32032	10.73	C_20_H_41_NO_2_	P	1.48	3.68E-02	0.82 ↓^a^	4.88E-01	1.12 ↑	4.43E-01	1.10 ↑
58	Typhasterol	447.34780	10.81	C_28_H_48_O_4_	N	1.82	2.81E-02	0.77 ↓^a^	9.79E-01	1.00 ↓	1.51E-01	1.21 ↑
59	Erucamide	338.34090	10.85	C_22_H_43_NO	P	1.92	9.36E-03	0.82 ↓^c^	5.65E-01	1.09 ↑	1.59E-01	1.07 ↑
60	Palmitic amide	256.26292	11.24	C_16_H_33_NO	P	2.24	4.46E-04	0.67 ↓^c^	8.43E-02	1.15 ↑	5.64E-03	1.24 ↑^c^

^a^ Adjusted-*p* values <0.05.

^b^Metabolites were identified by reference standards.

^c^ Adjusted-*p* values <0.01; Rt, retention time; VIP, variable importance in the projection obtained from APP/PS1 group vs. WT group; 12,13-DiHOME, 12,13-dihydroxy-9-octadecenoic acid; LysoPE, lysophosphatidylethanolamine; PS, phosphatidylserine; LysoPC, lysophosphatidylcholine; Cer, ceramide; P, positive ion mode; N, negative ion mode.

Subsequently, metabolic pathway enrichment analysis was performed to further investigate the potential pathogenesis of AD. These significantly altered metabolites in plasma of APP/PS1 mice were mainly involved in sphingolipid metabolism, glycerophospholipid metabolism, histidine metabolism, riboflavin metabolism, and taurine and hypotaurine metabolism (Figure 4C). Additionally, the correlation heatmap displays relationships between the 60 differential metabolites (Figure 4D). It was shown that the levels of unsaturated fatty amides and metabolites related to sphingolipid metabolism correlated negatively with saturated fatty amides and fatty acyl ethanolamides.

#### 3.3.3 Effect of GBLE treatment on the metabolic abnormality of APP/PS1 mice

The OPLS-DA models were also established for the comparisons between APP/PS1+Donepezil/GBLE group and APP/PS1 group based on the LC-MS/MS data derived from positive and negative ion mode, respectively (Figures 3F–I, Supplementary Figures S4C–F). Analogously, the score plots both revealed remarkable separations for the two comparisons. To probe the influence of GBLE on the metabolic aberration of AD, the metabolic signatures of WT, APP/PS1, APP/PS1+ Donepezil, and APP/PS1+GBLE groups were compared by focusing on the levels of the above differential metabolites. It was noteworthy that the vast majority of these 60 metabolites showed opposite trends for APP/PS1 vs*.* WT and APP/PS1+GBLE vs*.* APP/PS1, and 19 metabolites of which were significantly reversely regulated by GBLE, mainly including oleoyl ethanolamide, palmitic amide, brassylic acid, 5′-methylthioadenosine, ketosphingosine, and lysoPC(17:0) (Table 1). For the positive control group, 23 disordered metabolites were significantly reversed by donepezil. The effect of GBLE on regulating metabolic disturbance in APP/PS1 mice was similar to but slightly weaker than that of donepezil, and the regulated metabolites were somewhat different in the two groups. The HMDB classification for the obviously regulated metabolites was displayed in Figure 5A, and the area of each pie chart represented the relative proportion for each class of metabolites. This figure illustrated that most of the altered metabolites after GBLE intervention were classified as lipids and lipid-like molecules.

**FIGURE 6:**
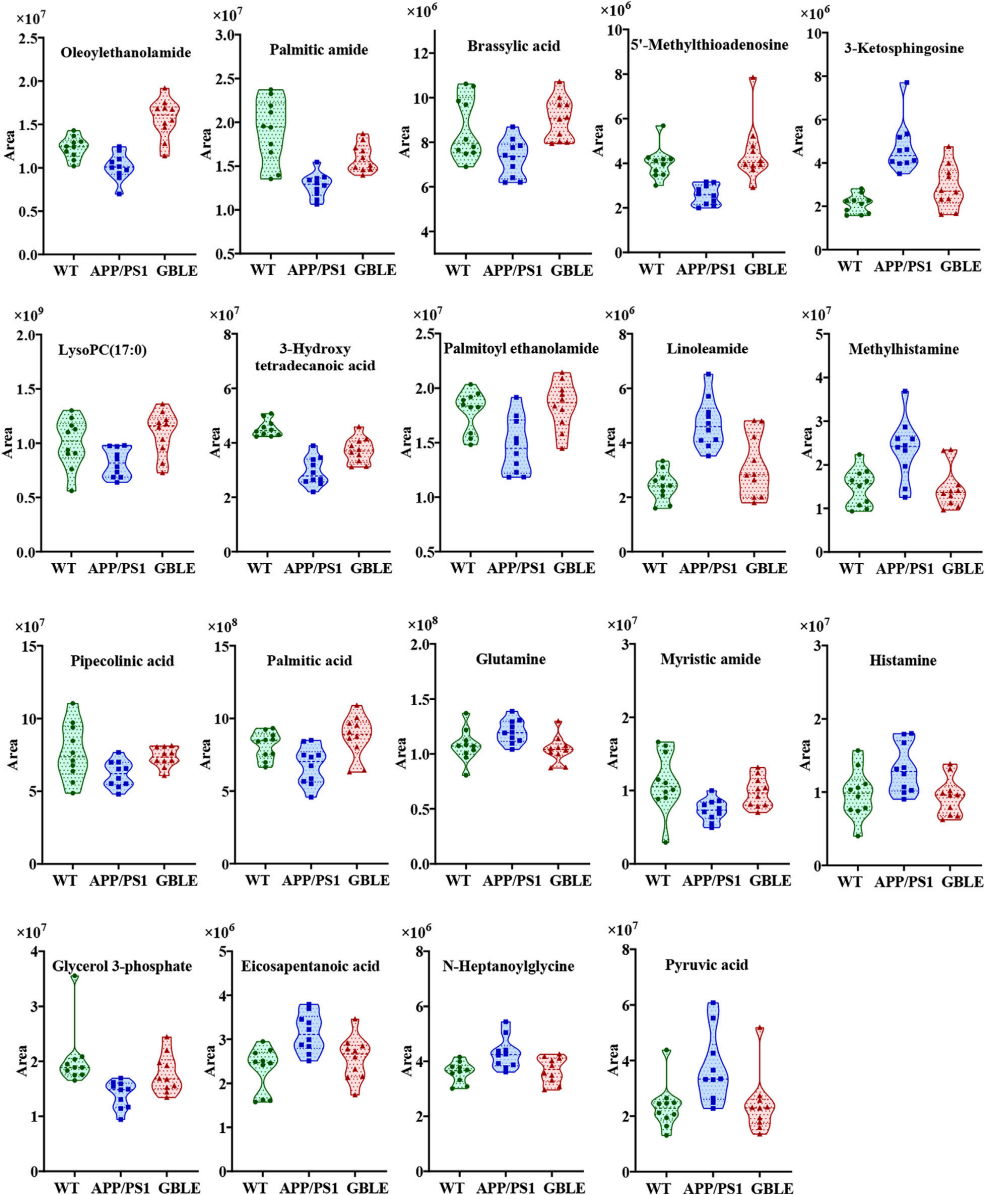
Violin plots showing the average peak area changes of each metabolite significantly ameliorated by GBLE.

The relative levels of these differential metabolites across different groups were visualized in a heat map based on the average of each group. As shown in Figure 5B, the color discrimination between APP/PS1 group and WT group was obvious, indicating the metabolic disturbance in AD. Most metabolites were reversibly regulated after GBLE and donepezil treatment as reflected by the change trends of colors. Furthermore, the violin plot visually exhibited the levels of 19 significantly altered metabolites in three different groups (Figure 6), and the impact of GBLE on the perturbed metabolites was evident. Metabolic pathway analysis was conducted to explore the underlying mechanism of GBLE in ameliorating AD. The 19 differential metabolites between APP/PS1+GBLE and APP/PS1 group were primarily associated with histidine metabolism, alanine, aspartate and glutamate metabolism, glycerophospholipid metabolism, glycerolipid metabolism, and pyruvate metabolism (Figure 4E). The impacts of major metabolic pathways were slightly different from that of donepezil treatment (Figure 4F).

### 3.4 Lipid signatures of APP/PS1 mice and the regulation effects of GBLE treatment on abnormal lipid metabolites

#### 3.4.1 Lipid identification

**FIGURE 7:**
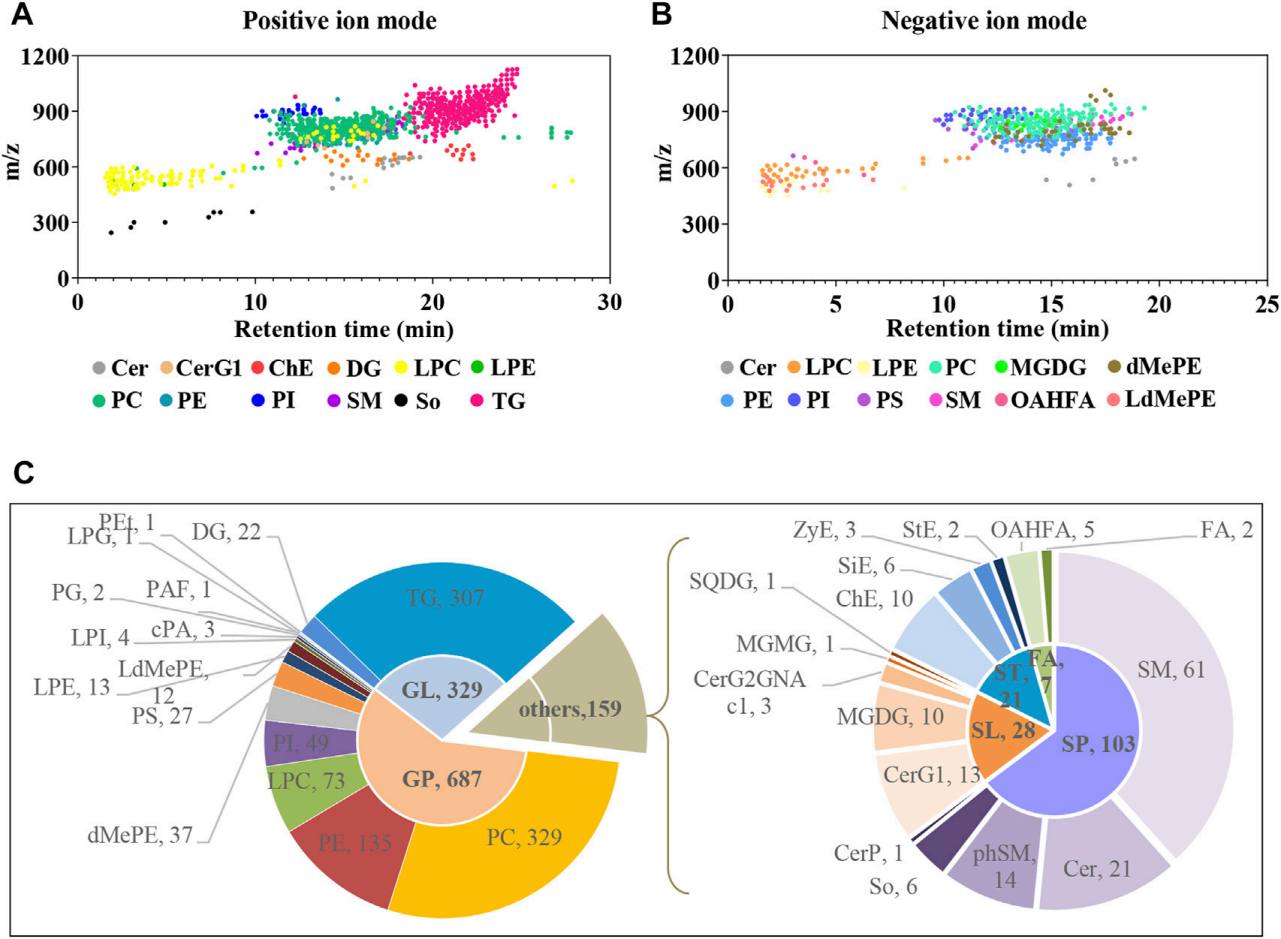
Delineation of plasma lipids detected by UHPLC-Q-Orbitrap HRMS. **(A,B)** Scatter plots of lipid features detected in positive and negative ion modes, respectively. **(C)** Pie charts showing the number of every lipid subclass belonging in each category. Cer, ceramides; CerG1, monogylcosylceramide; ChE, cholesteryl ester; DG, diglyceride; LPC, lysophosphatidylcholine; LPE, lysophosphatidylethanolamine; PC, phosphatidylcholine; PE, phosphatidylethanolamine; PI, phosphatidylinositol; SM, sphingomyelin; So, sphingoshine; TG, triglyceride; MGDG, monogalactosyldiacylglycerol; dMePE, dimethylphosphatidylethanolamine; PS, phosphatidylserine; OAHFA (O-acyl)-1-hydroxy fatty acid; LdMePE, Lysodimethylphosphatidylethanolamine; PG, phosphatidylglycerol; LPG, lysophosphatidylglycerol; PEt, phosphatidylethanol; PAF, platelet-activating factor; cPA, cyclic phosphatidic acid; SQDG, sulfoquinovosyldiacylglycerol; ZyE, zymosteryl; SiE, sitosteryl ester; MGMG, monogalactosylmonoacylglycerol; CerP, ceramides phosphate; phSM, phytosphingosine; StE, stigmasteryl ester; GP, glycerophospholipids; GL, glycerolipids; SP, sphingolipids; SL, saccharolipids; ST, sterol lipids; FA, fatty acyls.

Plasma untargeted metabolomic profiling suggested that the disordered metabolites and disturbed metabolic pathways in APP/PS1 mice mainly related to lipid metabolism. In line with this, the lipidomic analysis was further performed using UHPLC-Q-Orbitrap HRMS. In total, 1,116 and 513 lipid features covering varies subclasses were detected in positive and negative ion modes, respectively (Figure 7A and Figure 7B). After combining data derived from the two different ion modes and removing duplicated lipids, 1,175 lipid metabolites in six major categories were obtained, including 687 glycerophospholipids, 329 glycerolipids, 103 sphingolipids, 28 saccharolipids, 21 sterol lipids, and seven fatty acyls. Figure 7C delineates the number of every lipid subclass belonging in each category.

#### 3.4.2 Characterization of differential lipid metabolites in plasma samples between APP/PS1 and WT groups and the role of GBLE in regulation of lipids

**FIGURE 8:**
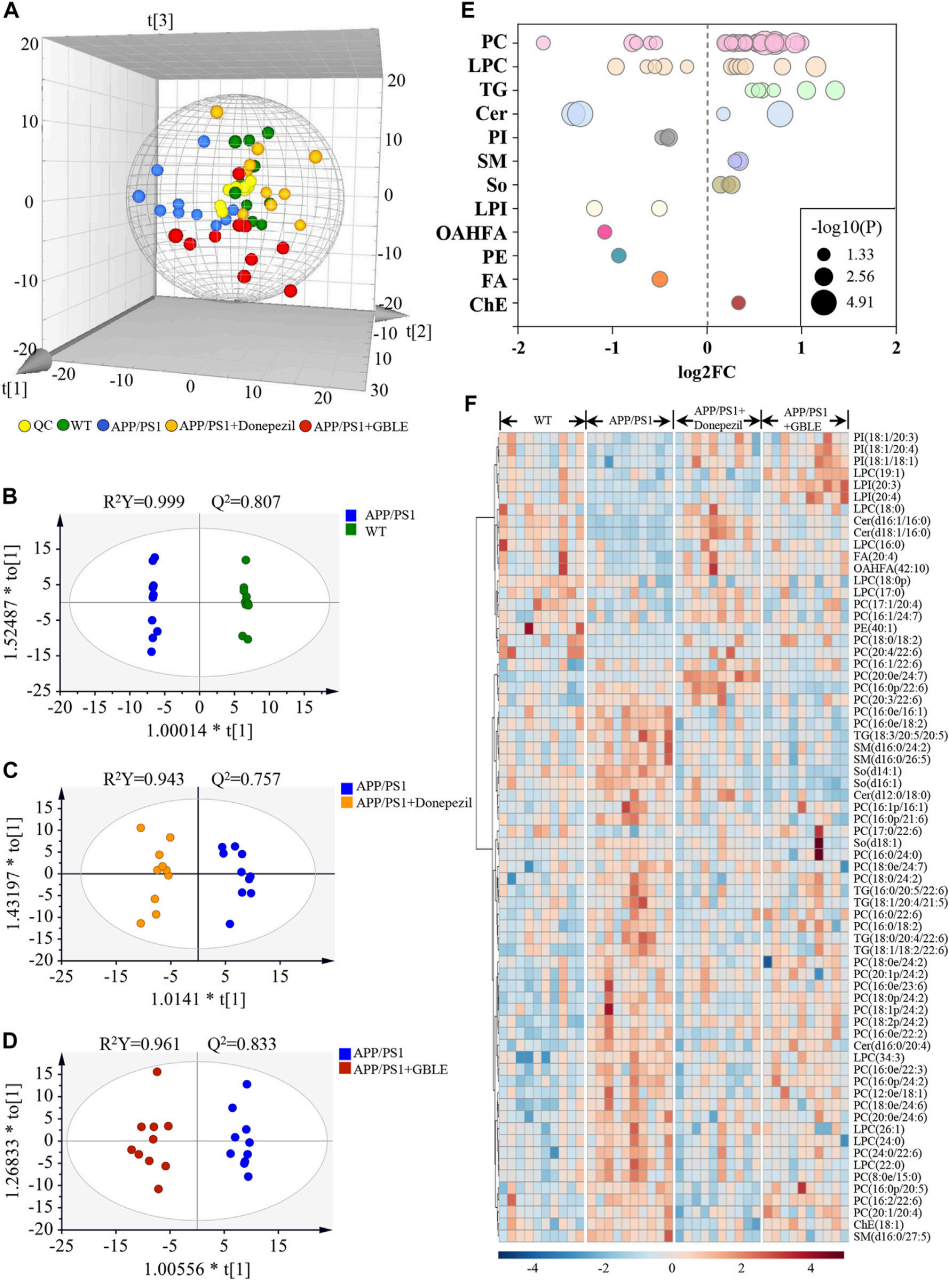
UHPLC-Q-Orbitrap HRMS-based lipidomic analysis**. (A)** Three dimensional PCA plot of all the plasma samples from different groups. **(B)** OPLS-DA score plot for APP/PS1 vs. WT group. **(C)** OPLS-DA score plot for APP/PS1+Donepezil vs. APP/PS1 group. **(D)** OPLS-DA score plot for APP/PS1+GBLE vs. APP/PS1 group. **(E)** Bubble chart of differential lipids for APP/PS1 vs. WT group. **(F)** Heatmap of the 68 differential lipids among the WT, APP/PS1, APP/PS1+Donepezil, and APP/PS1+GBLE groups. The colors from blue to red indicates the elevation of lipid metabolites.

The unsupervised PCA analysis was carried out, and there was a clear trend towards separation for APP/PS1 vs*.* WT group and APP/PS1+Donepezil/GBLE vs*.* APP/PS1 group as presented in the 3D score plot (Figure 8A). Additionally, it was noteworthy that QC samples were clustered in the center of the score plot, confirming the good reproducibility of instrument and high reliability of lipidomic data. Moreover, the OPLS-DA score plots showed complete separations between APP/PS1 and WT groups (R^2^Y = 0.999, Q^2^ = 0.807; Figure 8B), between APP/PS1+Donepezil and APP/PS1 groups (R^2^Y = 0.943, Q^2^ = 0.757; Figure 8C), and between APP/PS1+GBLE and APP/PS1 groups (R^2^Y = 0.961, Q^2^ = 0.833; Figure 8D).

The differential expressed lipid metabolites between APP/PS1 and WT mice were determined conforming to VIP scores >1.0 and adjusted-*p* values <0.05. Of the 68 altered lipids, 49 were significantly increased in APP/PS1 group mainly belonging to triglycerides (TG), lyso-phosphatidylcholines (LPC), phosphatidylcholines (PC), and sphingomyelins (SM) subclasses, while 19 were decreased mainly including phosphatidylinositols (PI), lyso-phosphatidylinositols (LPI), phosphatidylethanolamines (PE), and a fraction of PC and LPC (Figure 8E).

Taking the above disturbed lipid metabolites as the evaluation indices of therapeutic effect, the role of GBLE treatment on the plasma lipid fingerprint of AD was investigated through a comparison of APP/PS1+GBLE group vs*.* APP/PS1 group. As shown in Figure 8F, a number of differential lipids exhibited obvious recovery trend after treatment with GBLE. The effect of GBLE on lipid metabolism disorder in APP/PS1 mice was similar to but slightly weaker than that of donepezil. The result indicated that a lipid disorder occurred in APP/PS1 mice, and this abnormality could be ameliorated by GBLE administration. In general, GBLE showed significant effect on lipid metabolism in APP/PS1 mice.

## 4 Discussion

**FIGURE 9:**
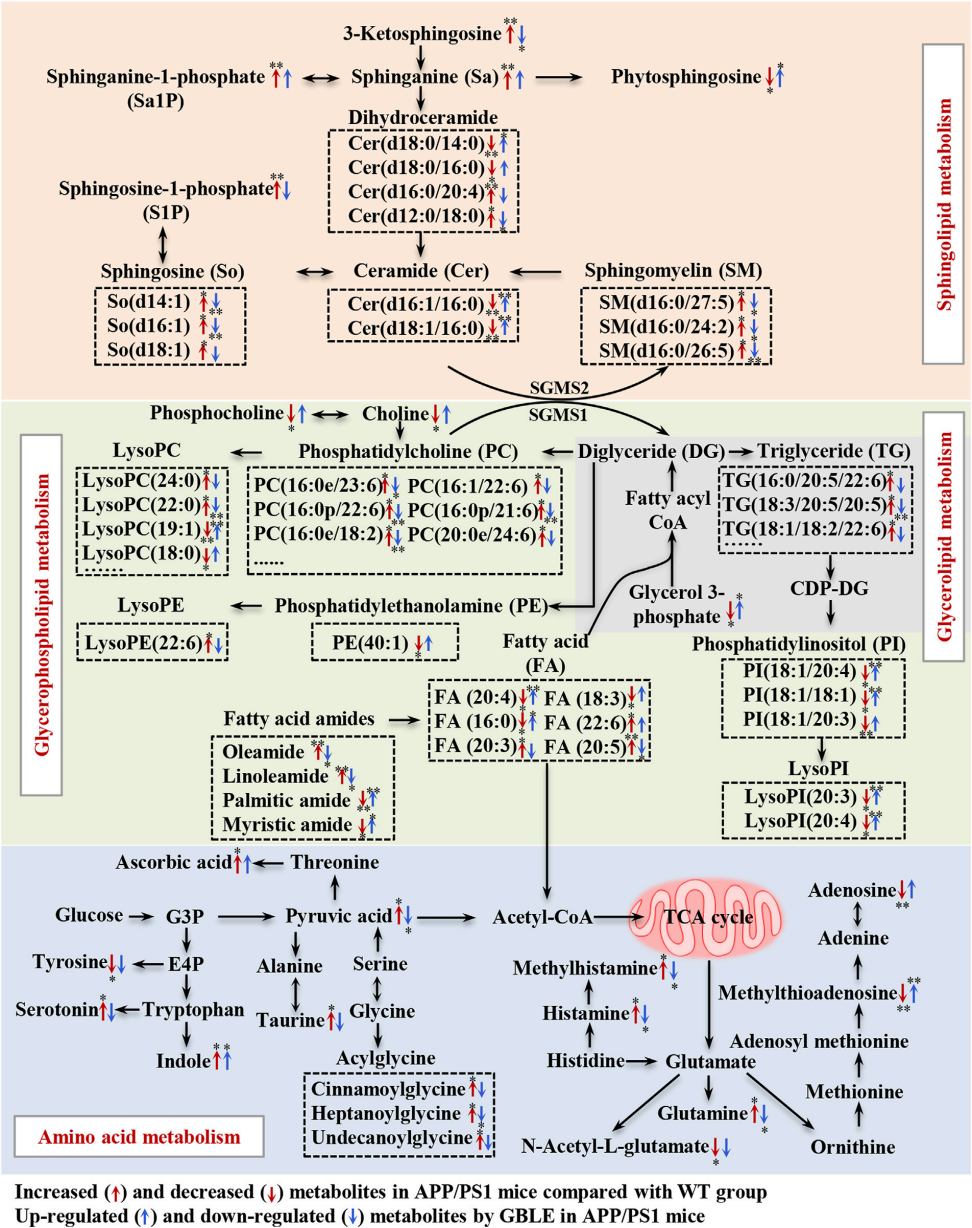
Disturbed metabolic pathway network in APP/PS1 mice and the interventional effects of GBLE by integrating metabolomics and lipidomics data. The red arrows represent altered metabolites in APP/PS1 mice compared with WT group, and the blue arrows represent the regulated metabolites by GBLE treatment. ^*^: adjusted-*p* < 0.05, ^**^: adjusted-*p* < 0.01.

In the present study, an integrated metabolomics and lipidomics approach was adopted to characterize the metabolomic phenotype of APP/PS1 mice and describe the metabolic fingerprint change after GBLE intervention. Taking together all the data, here, we delineated a metabolic correlation network based on the differential metabolites and lipids, and proposed several significant metabolic pathways in response to GBLE treatment for APP/PS1 mice (Figure 9). The major findings of this work are summarized as follows: 1) Administration of GBLE significantly improved the cognitive function and alleviated Aβ deposition in APP/PS1 mice. 2) The plasma metabolic signature of APP/PS1 mice was primarily characterized by disrupted sphingolipid metabolism, glycerophospholipid metabolism, glycerolipid metabolism, and amino acid metabolism. 3) GBLE treatment could reverse the disturbance of many key endogenous metabolites and regulate abnormal metabolism in APP/PS1 mice, particularly lipid dishomeostasis. The results may be helpful in understanding the potential pathogenesis of AD and shed new light on the therapeutic mechanism of GBLE in the treatment of AD.

In recent years, metabolomics has been widely employed to characterize the metabolic alterations of diseases and provides a potential approach to explore the complex action modes of TCMs. During the past 10 years, over hundreds of metabolomics studies have been conducted to uncover the metabolic changes in multiple biological matrices including plasma, serum, cerebrospinal fluid, urine, feces, and tissues of different AD animal models and patients (González-Domínguez et al., 2018; Dai et al., 2022). These studies demonstrated disturbances of energy-related metabolism, fatty acid metabolism, nitrogen metabolism, amino acid metabolism, and many others related to AD. Similarly to previous studies, significant alterations were observed in levels of glycerophospholipids, sphingolipids, glycerolipids, fatty acids, amino acids, and nucleotides in the present work. Lipidomics analysis showed significant changes in the levels of PCs, LPCs, PEs, Cers, SMs and some other lipids, corroborating the pivotal role of lipid metabolism in the pathogenesis of AD, despite not entirely consistent change with previous literatures on AD-related lipidome and phospholipidome (González-Domínguez et al., 2014a; González-Domínguez et al., 2014b).

Lipids, the essential components of cellular membranes, play crucial roles in many biological processes, such as maintenance of cell structure and function, energy storage, signal transduction and regulation of gene expression (Brügger, 2014; Han, 2016). The lipids have been classified into eight categories: fatty acyls, glycerolipids, glycerophospholipids, sphingolipids, sterol lipids, saccharolipids, prenol lipids, and polyketides (Fahy et al., 2005), and each category consists of further lipid subclasses. Among these lipids, glycerophospholipids, sphingolipids, and cholesterol are mainly localized in neuronal membranes and myelin (Wang et al., 2021). Abnormal lipid metabolism is closely correlated with neurological diseases and affects cognitive function (Wong et al., 2017). It is widely accepted that the accumulation of Aβ is strongly associated with cognitive dysfunction of AD. Lipid rafts, enriched in sphingolipids and glycerophospholipids, not only promote the generation of Aβ peptides and facilitate the formation of toxic oligomers, but also host specific neuronal receptors which participate in the signal transduction of neuropathological events. A wealth of evidence has unambiguously linked lipid rafts to neurodegenerative diseases, including AD (Mesa-Herrera et al., 2019). Previous studies have shown significant alterations in molecular structure and functionality of lipid rafts in the frontal cortex of brains in AD patients (Rushworth and Hooper, 2010). Therefore, we speculated that the metabolic disorder of sphingolipids and glycerophospholipids induced the abnormal expression of Aβ *via* breaking down the lipid rafts homeostasis. Nevertheless, it is still no consensus on the change trends of various lipids for AD from previous studies.

In this work, one of the most changes was observed in sphingolipid metabolism in the plasma of APP/PS1 mice. Compared with WT mice, the levels of sphingosine, sphinganine, 3-ketosphingosine, sphingosine-1-phosphate (S1P), sphinganine-1-phosphate and sphingomyelins (SM) were significantly increased in APP/PS1 mice, while ceramides presented both negative and positive associations with AD. Ceramides, with the structure containing a long-chain sphingoid base and one N-acylated fatty acid, are central molecules in the biosynthesis and catabolism of sphingolipids (Pralhada Rao et al., 2013). According to the fatty acids contained in the structure, previous study demonstrated that the increase of ceramides containing very long fatty acids was more pronounced in AD (Cutler et al., 2004), which was associated with the elevated expression of long-chain ceramide synthase (Katsel et al., 2007). It was corroborated by the increased level of Cer (d16:0/20:4) in APP/PS1 mice in this work. Some previous researches have demonstrated that upregulated ceramides can promote aggregation of Aβ through the effect on lipid rafts (Badawy et al., 2018). On the other hand, the increased level of unsaturated fatty acid-containing ceramide (Cer d16:0/20:4) together with a significant decrease in several saturated fatty acids-containing ceramides (Cer d16:1/16:0, Cer d18:1/16:0, Cer d18:0/14:0, and Cer d18:0/16:0) were observed in APP/PS1 mice. The results could be consequent with the elevated activity of stearoyl-CoA desaturase, which was associated with AD (Astarita et al., 2011). Our results were consistent with a recent study (Huynh et al., 2020). In addition, SM may be another specific biomarker for AD, which increased the risk of AD in previous clinical studies (Varma et al., 2018). Toledo reported that increase in levels of SMs in AD patients was related to cognitive decline and brain atrophy (Toledo et al., 2017). Kosicek found significantly increased SMs levels in the cerebrospinal fluid in patients with AD compared with healthy controls (Kosicek et al., 2012). In accordance with this, here we showed that APP/PS1 mice displayed elevated levels of SM(d16:0/27:5), SM(d16:0/24:2), and SM(d16:0/26:5) in plasma. S1P, a signaling molecule, is produced by ceramides *via* ceramidase and subsequent sphingosine kinase. Previous studies have reported that ceramidase is up-regulated in AD (He et al., 2010), as well as the activity of sphingosine kinase 2 (Takasugi et al., 2011), which could be responsible for the significantly increased levels of S1P in APP/PS1 mice in the present study. Excitingly, our results suggested that GBLE showed significant improvement in the disturbed sphingolipid metabolism, and sphingolipids may serve as putative therapeutic targets of GBLE on AD.

The results also suggested significant alterations in glycerophospholipids and glycerolipids for APP/PS1 mice, such as PCs, LPCs, PEs, LPEs, PIs, LPIs, and TGs. Glycerol 3-phosphate, a key molecule in the initial step of glycerolipid and glycerophospholipids metabolism, was significantly lower in APP/PS1 mice when compared with WT group, which is in agreement with a recent research (Watanabe et al., 2021). Previously published researches supported possible associations between glycerophospholipids and amyloid deposits related to pathology of AD (Whiley et al., 2014). Huo et al. discovered that increased levels of some PCs were related to cognitive impairment (Huo et al., 2020). Toledo reported that the levels of PCs were elevated in the cerebrospinal fluid of AD patients and associated with aberrant Aβ1-42 (Toledo et al., 2017). Similar to these results, most PCs were upregulated in the plasma of APP/PS1 mice in the present study. In fact, researches on the levels of PCs in AD were not uniform and the results were somewhat controversial (Toledo et al., 2017; Huo et al., 2020; Bhawal et al., 2021). These contrary findings could arise by diverse functions of different PCs, which can be responsible for the minority of decreased PCs in our study. Our results implicated that specific PCs, rather than PCs as a whole, might play important roles in AD. LPCs serve as mediators in multiple neuronal pathways involved in neurobiology of AD (Frisardi et al., 2011), and previous studies have reported decreased levels of LPCs in AD (González-Domínguez et al., 2014b). However, in the present work, it was observed that the levels of LPCs containing fatty acids with carbon atoms <20 were decreased, while those with carbon atoms >20 were increased in the plasma of APP/PS1 mice, which might be worthy to be studied further. In addition, numerous studies reported reduced levels of PEs (Igarashi et al., 2011; González-Domínguez et al., 2014b), as confirmed in our data. As for glycerolipids, the levels of TGs were markedly increased in APP/PS1 mice, and our findings were consistent with a related study in which a higher level of TG was observed in the serum of AD patients (Berezhnoy et al., 2022). Notably, most of these changes showed a significant correction after GBLE administration. The close relationship between these molecules and AD may provide a new strategy for further research on the treatment of this disease.

Besides, we observed a dramatic disorder in the fatty amides levels in the plasma of APP/PS1 mice. In a recent study on AD biomarker discovery, the plasma fatty amides were demonstrated to be associated with brain amyloid burden, hippocampal volume, and memory (Kim et al., 2019). Palmitic amide is a primary fatty acid amide and plays a pivotal role in cellular signal transduction. Decreased serum palmitic amide in AD was reported earlier (Chu et al., 2016). Oleamide has been early identified in the brains of sleep-deprived mice and cats (Cravatt et al., 1995) and is a vital regulatory lipid in central nervous system. Kim M. et al. Identified significantly higher levels of oleamide and linoleamide in the plasma of AD patients (Kim et al., 2019). These findings agreed with our results, and we found that the saturated fatty amides seemed decreased while unsaturated fatty amides were increased in APP/PS1 mice. The opposite directions could be attributed to increased desaturase activity in AD. Indeed, so far very little is known about the biological function of fatty amides, which may be key therapeutical targets in the treatment of AD.

Additionally, the APP/PS1 mice showed perturbed amino acid metabolism, tricarboxylic acid (TCA) cycle and fatty acids oxidation, and these alterations were ameliorated following 3-month GBLE administration. The dyshomeostasis of aromatic amino acids and the synthesis of neurotransmitters was associated with AD (González-Domínguez et al., 2021), which was corroborated by the altered tyrosine and serotonin in the present work. Histamine and methylhistamine were significantly elevated in APP/PS1 mice, and previous evidence suggested that neurotransmitter systems including neuronal histamine could contribute to the development and maintenance of AD-related cognitive deficits (Zlomuzica et al., 2016). Acylglycines are produced through the action of glycine N-acyltransferase and they are normally minor metabolites of fatty acids. Elevated levels of acylglycines (cinnamoylglycine, heptanoylglycine, and undecanoylglycine) appeared in the plasma of APP/PS1 mice, indicating a disordered fatty acid oxidation in AD. The increased level of pyruvate was related to dysregulated TCA cycle, which was consistent with previous studies (Rushworth and Hooper, 2010). In this work, we also found APP/PS1 mice displayed a declined level of acylcarnitine, which was not merely a cofactor in β-oxidation, but also had beneficial effects in the treatment of neurological diseases (Jones et al., 2010). Various studies have reported important deregulations in the metabolism of purines and pyrimidines (González-Domínguez et al., 2021). Adenosine, an important neuroprotective factor (Rahman, 2009), was dramatically decreased in APP/PS1 mice and upregulated by GBLE intervention, as well as methylthioadenosine.

Of note, in addition to AD, GBLE is also used for prevention and therapy of myocardial ischemia. A metabolomics study demonstrated that the cardioprotective effect of GBLE was achieved through comprehensive regulation of multiple metabolic pathways covering lipid, energy, amino acid and nucleotide metabolism (Wang et al., 2016). The similar regulating effect of GBLE on different diseases seems to reflect the strategy of “homotherapy for heteropathy” for TCM.

To sum up, we comprehensively described the metabolic signatures of AD and evaluated the effects of GBLE on plasma metabolome and lipidome in APP/PS1 mice. However, this work has some limitations worth noting. Firstly, the metabolomic and lipidomic profiles were obtained only based on the plasma samples. Characterizing the metabolic signatures of brain tissues is needed in future studies, and a comprehensive research through combining the data from plasma and brain tissue is crucial for unveiling the pathological mechanisms of AD and therapeutic targets of GBLE. Secondly, we observed that numerous endogenous metabolites were regulated by GBLE. However, the molecular mechanisms are still not well understood. It is essential to further explore the causality and investigate the biological context in which theses metabolites operate by integrating additional information such as protein and gene expression in the future. Besides, this study only includes male mice, and it is uncertain whether the effect of GBLE is the same in female mice. Nevertheless, this study provides a better understanding on the metabolism-based neuroprotective effects of GBLE on AD.

## 5 Conclusion

In conclusion, we delineated the metabolic disturbance in the plasma of APP/PS1 mice and investigated the overall therapeutic effect of GBLE on AD through UHPLC-MS/MS-based metabolomic and lipidomic approach. The metabolic perturbation in APP/PS1 mice was primarily related to sphingolipid metabolism, glycerophospholipid metabolism, glycerolipid metabolism, and amino acid metabolism, and this dyshomeostasis could be ameliorated by GBLE treatment. These results deepen our knowledge about the pathophysiological mechanisms of AD, provide available evidence for the neuroprotective effect of GBLE administration, and reveal the characteristic of multiple components, targets and pathways for traditional Chinese medicines.

## Data Availability

The original contributions presented in the study are included in the article/Supplementary Materials, further inquiries can be directed to the corresponding author.

## Supplementary material

Supplementary Figure S1 The animal experiment schedule.

Supplementary Figure S2 The total ion chromatograms of GBLE in **(A)** positive ion mode and **(B)** negative ion mode.

Supplementary Figure S3 Reliability assessment of the analytical method by the performance of QC samples and internal standard responses. **(A,B)** PCA score plots of all the samples in positive and negative ion modes. **(C,D)** Relative standard deviation (RSD) distribution of metabolic ion features for QC samples detected in positive and negative ion modes. **(E,F)** Relative abundance variabilities of 2-chloro-L-phenylalanine (ESI^+^ internal standard) and ketoprofen (ESI^-^ internal standard) with respect to the injection order.

Supplementary Figure S4 The OPLS-DA score plots and corresponding permutation tests for **(A,B)** APP/PS1 *vs.* WT, **(C,D)** APP/PS1+Donepezil *vs*. APP/PS1 and **(E,F)** APP/PS1+GBLE *vs*. APP/PS1 in negative ion mode.
