# Supplementary material for: Comprehensive metabolomics and lipidomics profiling uncovering neuroprotective effects of Ginkgo biloba L. leaf extract on Alzheimer’s disease
Source: Front Pharmacol. 2022 Dec 21;13:1076960. doi: 10.3389/fphar.2022.1076960 (PMC9810818; doi:10.3389/fphar.2022.1076960)
Supplement: Supplementary file 1 [file Table1.DOCX]

**Supplementary Table S1** The identified chemical constituents of *Ginkgo biloba* L. leaf extract.

| **No.** | **Compounds** | **t_R_ (min)** | **Formula** | **Ion mode** | **ES/expected (m/z)** | **ES/measured (m/z)** | **Delta (ppm)** | **Fragment Ions (m/z)** | **Classfication** |
| --- | --- | --- | --- | --- | --- | --- | --- | --- | --- |
| 1 | Quinic acid^*^ | 1.20 | C7H12O6 | N | 191.05611 | 191.05565 | -2.415 | 93.03, 85.03 | Carboxylic acids |
| 2 | 3-Hydroxybenzoic acid | 1.24 | C7H6O3 | N | 137.02441 | 137.02417 | -1.805 | 93.03 | Carboxylic acids |
| 3 | 3-Hydroxybenzoic acid isomer | 1.24 | C7H6O3 | N | 137.02441 | 137.02412 | -2.170 | 109.03, 93.03 | Carboxylic acids |
| 4 | Isocitric acid | 1.50 | C6H8O7 | N | 191.01972 | 191.05594 | -0.897 | 111.05, 93.03, 85.03, 73.03, 67.02 | Carboxylic acids |
| 5 | p-Coumaroylquinic acid | 1.90 | C16H18O8 | N | 337.09289 | 337.09323 | 1.007 | 173.06, 163.04, 145.07, 119.05, 93.03 | Carboxylic acids |
| 6 | Gallic acid^*^ | 1.92 | C7H6O5 | N | 169.01424 | 169.01399 | -1.518 | 125.02 | Carboxylic acids |
| 7 | Ethyl gallate | 2.02 | C9H10O5 | P | 199.06009 | 199.06027 | 0.854 | 153.02, 135.04, 97.06 | Others |
| 8 | Protocatechuic aldehyde-Glc | 2.03 | C13H6O8 | N | 299.07724 | 299.07712 | -0.403 | 137.02, 93.03 | Others |
| 9 | 6-Hydroxykynurenic acid | 3.19 | C10H7NO4 | N | 204.03023 | 204.02997 | -1.279 | 159.03, 131.04, 90.04 | Carboxylic acids |
| 10 | Protocatechuic acid^*^ | 3.43 | C7H6O4 | N | 153.01933 | 153.01906 | -1.777 | 109.03, 91.02, 81.03 | Carboxylic acids |
| 11 | Protocatechuic aldehyde | 5.96 | C7H6O3 | N | 137.02441 | 137.02412 | -2.170 | 119.01, 109.03, 108.02, 93.03, 91.02 | Others |
| 12 | Catechin^*^ | 7.45 | C15H14O6 | P | 291.08631 | 291.08582 | -1.699 | 165.05, 161.06, 147.04, 139.04, 123.04 | Flavonoids |
| 13 | Caffeic acid^*^ | 9.30 | C9H8O4 | N | 179.03498 | 179.03485 | -0.737 | 135.05, 117.03, 107.05, 89.04 | Carboxylic acids |
| 14 | Bilobalide isomer | 9.45 | C15H18O8 | N | 325.09289 | 325.09277 | -0.371 | 163.04, 119.05 | Terpenoids |
| 15 | Protocatechuic aldehyde isomer | 9.76 | C7H6O3 | N | 137.02441 | 137.02412 | -2.170 | 109.03, 93.03 | Others |
| 16 | Quercetin-3-O-rhamnosyl-hexoside-7-O-glc isomer | 13.22 | C33H40O21 | N | 771.19893 | 771.19800 | -1.207 | 462.08, 301.04, 299.02, 271.03, 179.00, 151.00, 107.01 | Flavonoids |
| 17 | Ginkgolide P | 13.79 | C20H24O10 | N | 423.12967 | 423.12979 | 0.283 | 367.14, 349.13, 287.13, 177.06, 113.02, 101.02, 72.99 | Terpenoids |
| 18 | Ginkgolide Q isomer | 15.49 | C20H24O11 | N | 439.12458 | 439.12448 | -0.238 | 383.13, 365.12, 321.13, 303.12, 259.13, 141.02, 125.02, 72.99 | Terpenoids |
| 19 | Ginkgolide J | 16.00 | C20H24O10 | N | 423.12967 | 423.12976 | 0.212 | 305.14, 243.14, 185.06, 125.02, 101.02, 72.99 | Terpenoids |
| 20 | Quercetin-3-O-Glc-7-O-Rha | 16.23 | C27H30O16 | N | 609.14610 | 609.14587 | -0.390 | 463.09, 447.09, 446.09, 301.04, 299.02 | Flavonoids |
| 21 | Bilobalide^*^ | 16.42 | C15H18O8 | N | 325.09289 | 325.09277 | -0.371 | 251.09, 165.13, 163.11, 119.09, 101.06 | Terpenoids |
| 22 | Quercetin-3-O-rhamnosyl-hexoside-7-O-glc | 16.81 | C33H40O21 | N | 771.19893 | 771.19763 | -1.687 | 462.08, 301.04, 299.02, 271.03, 179.00, 151.00, 107.01 | Flavonoids |
| 23 | Ferulic acid | 17.14 | C10H10O4 | P | 195.06518 | 195.06502 | -0.848 | 177.05, 145.03, 117.03, 89.04 | Carboxylic acids |
| 24 | Isorhamnetin-3-O-rhamnosylhexoside-7-O-Glc | 18.29 | C34H42O21 | N | 785.21458 | 785.21381 | -0.982 | 623.16, 315.05, 300.03, 271.02, 243.03, 151.00, 107.01 | Flavonoids |
| 25 | Myricetin-7-Oglucosyl-Rha | 19.74 | C27H30O17 | N | 625.14102 | 625.14081 | -0.340 | 317.03, 179.00, 151.00, 137.02 | Flavonoids |
| 26 | Ginkgolide Q | 21.81 | C20H24O11 | N | 439.12458 | 439.12442 | -0.375 | 383.13, 365.12, 321.13, 303.12, 259.13, 141.02, 125.02, 72.99 | Terpenoids |
| 27 | Rutin isomer | 22.15 | C27H30O16 | N | 609.14610 | 609.14618 | 0.118 | 300.03, 271.02, 255.03, 151.00 | Flavonoids |
| 28 | Ginkgolide C^*^ | 22.99 | C20H24O11 | N | 439.12458 | 439.12430 | -0.648 | 383.13, 365.12, 321.13, 303.12, 259.13, 141.02, 125.02, 72.99 | Terpenoids |
| 29 | Quercetin-3-O-[6-rhamnosyl-2-(6-p-coumaroylglucosyl)]-Glc | 23.18 | C42H46O23 | N | 917.23571 | 917.23395 | -1.919 | 462.08, 299.02, 179.00, 151.00, 107.01 | Flavonoids |
| 30 | Rutin^*^ | 23.63 | C27H30O16 | N | 609.14610 | 609.14600 | -0.177 | 300.03, 271.02, 255.03, 151.00 | Flavonoids |
| 31 | Isorhamnetin-3-O-(2,6-dirhamnosyl)-Glc | 23.97 | C34H42O20 | N | 769.21966 | 769.21875 | -1.192 | 315.05, 314.04, 299.02, 271.02, 243.03, 151.00 | Flavonoids |
| 32 | Quercetin-3-O-glucoside | 24.15 | C21H20O12 | N | 463.08819 | 463.08826 | 0.131 | 302.04, 301.04, 151.00 | Flavonoids |
| 33 | Luteolin-7-O-Glc^*^ | 24.98 | C21H20O11 | N | 447.09328 | 447.09314 | -0.323 | 285.04, 256.04, 227.04, 151.00, 133.03 | Flavonoids |
| 34 | Kaempferol-3-O-[6-rhamnosyl-2-(6-p-coumaroylglucosyl)]-Glc | 25.17 | C42H46O22 | N | 901.24079 | 901.23969 | -1.227 | 739.19, 285.04, 284.03, 227.04, 211.04 | Flavonoids |
| 35 | Isorhamnetin-3-O-Glc isomer | 25.71 | C22H22O12 | N | 477.10384 | 477.10388 | 0.064 | 315.05, 314.04, 299.02, 271.02, 243.03, 227.03, 151.00 | Flavonoids |
| 36 | Quercetin-3-O-glucoside isomer | 26.08 | C21H20O12 | N | 463.08819 | 463.08841 | 0.455 | 302.04, 301.04, 151.00 | Flavonoids |
| 37 | Rutin isomer | 26.66 | C27H30O16 | N | 609.14610 | 609.14624 | 0.217 | 300.03, 271.02, 255.03, 151.00 | Flavonoids |
| 38 | kaempferol 3-O-rutinoside^*^ | 26.87 | C27H30O15 | N | 593.15119 | 593.15125 | 1.020 | 285.04, 255.03, 227.04, 151.00 | Flavonoids |
| 39 | Luteolin-7-O-Glc isomer | 27.58 | C21H20O11 | N | 447.09328 | 447.09323 | -0.122 | 285.04, 256.04, 227.04, 151.00, 133.03 | Flavonoids |
| 40 | Isorhamnetin-3-O-(2-glucosyl)-Rha | 27.76 | C28H32O16 | N | 623.16175 | 623.16156 | -0.318 | 315.05, 300.03, 271.02, 243.03, 151.00, 107.01 | Flavonoids |
| 41 | Syringetin-3-O-glucosyl-Rha | 28.27 | C29H34O17 | N | 653.17232 | 653.17230 | -0.035 | 345.06, 329.03, 301.03, 286.01, 273.04, 258.02, 242.02 | Flavonoids |
| 42 | Myricetin^*^ | 28.28 | C15H10O8 | N | 317.03029 | 317.02988 | -1.295 | 179.00, 151.00, 137.02, 109.03 | Flavonoids |
| 43 | Isorhamnetin-3-O-Glc | 28.48 | C22H22O12 | N | 477.10384 | 477.10382 | -0.050 | 315.05, 314.04, 299.02, 285.04, 271.02, 257.05, 243.03, 151.00 | Flavonoids |
| 44 | Apigenin 7-O-glucoside^*^ | 28.59 | C21H20O10 | N | 431.09837 | 431.09830 | -0.162 | 269.04, 211.04, 151.00, 117.03 | Flavonoids |
| 45 | Syringetin-3-O-glucosyl-Rha isomer | 29.48 | C29H34O17 | N | 653.17232 | 653.17236 | 0.057 | 345.06, 329.03, 301.04, 286.01, 258.02 | Flavonoids |
| 46 | Kaempferol 3-O-rutinoside isomer | 29.79 | C27H30O15 | N | 593.15119 | 593.15118 | -0.022 | 285.04, 255.03, 227.04, 151.00 | Flavonoids |
| 47 | Quercetin isomer | 29.85 | C15H10O7 | N | 301.03537 | 301.03522 | -0.518 | 151.00, 149.02, 107.01 | Flavonoids |
| 48 | Ginkgolide M | 30.51 | C20H24O10 | N | 423.12967 | 423.12991 | 0.567 | 367.14, 349.13, 243.14, 186.07, 127.04, 115.04, 72.99 | Terpenoids |
| 49 | Luteolin-7-O-Glc isomer | 30.65 | C21H20O11 | N | 447.09328 | 447.09320 | -0.189 | 285.04, 256.04, 227.04, 151.00, 133.03 | Flavonoids |
| 50 | Isorhamnetin-3-O-Glc isomer | 30.74 | C22H22O12 | N | 477.10384 | 477.10376 | -0.187 | 315.05, 314.04, 300.03, 272.03, 243.03, 227.04, 151.00 | Flavonoids |
| 51 | Apigenin 7-O-glucoside isomer | 31.32 | C21H20O10 | N | 431.09837 | 431.09814 | -0.533 | 269.04, 211.04, 151.00, 132.02 | Flavonoids |
| 52 | Ginkgolide B isomer | 31.46 | C20H24O10 | N | 423.12967 | 423.12970 | 0.071 | 367.14, 349.13, 305.14, 143.03, 141.02, 125.02, 113.02, 72.99 | Terpenoids |
| 53 | Ginkgolide A^*^ | 31.99 | C20H24O9 | N | 407.13475 | 407.13449 | -0.652 | 363.15, 351.15, 335.15, 273.15, 247.17, 229.16, 135.08 | Terpenoids |
| 54 | Ginkgolide B^*^ | 32.27 | C20H24O10 | N | 423.12967 | 423.12964 | -0.071 | 367.14, 143.03, 141.02, 125.02, 113.02, 72.99 | Terpenoids |
| 55 | Kaempferol-3-O-(2,6-dirhamnosyl)-Glc | 34.52 | C36H36O17 | N | 739.18797 | 739.18762 | -0.477 | 285.04, 284.03, 227.04, 211.04, 151.00 | Flavonoids |
| 56 | Myricetin isomer | 34.73 | C15H10O8 | N | 317.03029 | 317.02994 | -1.105 | 179.00, 151.00, 137.02, 109.03 | Flavonoids |
| 57 | Quercetin^*^ | 34.77 | C15H10O7 | N | 301.03537 | 301.03500 | -1.249 | 179.00, 151.00, 149.02, 107.01 | Flavonoids |
| 58 | Luteolin^*^ | 34.88 | C15H10O6 | N | 285.04046 | 285.04041 | -0.180 | 199.04, 151.00, 149.02, 134.03, 133.03, 107.01 | Flavonoids |
| 59 | Isorhamnetin isomer | 35.54 | C16H12O7 | N | 315.05102 | 315.05109 | 0.203 | 300.03, 271.02, 243.03, 151.00, 107.01 | Flavonoids |
| 60 | Flavanonol | 36.30 | C15H12O3 | P | 241.08592 | 241.08531 | -2.534 | 147.04, 121.03 | Flavonoids |
| 61 | Apigenin^*^ | 38.29 | C15H10O5 | N | 269.04554 | 269.04547 | -0.285 | 225.06, 151.00, 117.03, 107.01 | Flavonoids |
| 62 | Quercetin isomer | 38.84 | C15H10O7 | N | 301.03537 | 301.03519 | -0.617 | 179.00, 151.00, 107.01 | Flavonoids |
| 63 | Kaempferol^*^ | 38.85 | C15H10O6 | N | 285.04046 | 285.04037 | -0.320 | 229.05, 211.04, 185.06, 159.04, 117.03 | Flavonoids |
| 64 | Ginkgolide A isomer | 39.70 | C20H24O9 | N | 407.13475 | 407.13467 | -0.210 | 351.15, 333.13, 245.15, 151.08, 127.04 | Terpenoids |
| 65 | Isorhamnetin^*^ | 39.73 | C16H12O7 | N | 315.05102 | 315.05096 | -0.209 | 300.03, 271.02, 243.03, 151.00, 107.01 | Flavonoids |
| 66 | Bilobetin-7-O-Glc isomer | 40.14 | C37H30O15 | P | 715.16574 | 715.16357 | -3.043 | 553.11, 435.07, 403.04, 297.07, 153.02, 121.03 | Flavonoids |
| 67 | Bilobetin-7-O-Glc | 40.88 | C37H30O15 | P | 715.16574 | 715.16394 | -2.526 | 553.11, 435.07, 403.04, 297.07, 153.02, 121.03 | Flavonoids |
| 68 | Ginkgolide K | 41.44 | C20H22O9 | N | 405.11910 | 405.11893 | -0.433 | 349.13, 287.13, 125.02, 72.99 | Terpenoids |
| 69 | Amentoflavone^*^ | 43.11 | C30H18O10 | P | 539.09727 | 539.09637 | -1.675 | 497.09, 421.05, 403.04, 387.08, 377.06, 335.05 | Flavonoids |
| 70 | Isoginkgetin-7-O-Glc | 44.16 | C38H32O15 | P | 729.18139 | 729.17969 | -2.340 | 567.13, 417.06, 167.03, 121.03 | Flavonoids |
| 71 | Ginkgetin-7-O-Glc | 44.71 | C38H32O15 | P | 729.18139 | 729.17932 | -2.848 | 567.13, 417.06, 167.03, 121.03 | Flavonoids |
| 72 | Ginkgetin-7-O-Glc isomer | 45.57 | C38H32O15 | P | 729.18139 | 729.17957 | -2.505 | 567.13, 135.04 | Flavonoids |
| 73 | Bilobetin | 46.05 | C31H20O10 | P | 553.11292 | 553.11194 | -1.778 | 435.07, 403.04, 297.08, 153.02, 121.03 | Flavonoids |
| 74 | Ginkgetin-7-O-Glc isomer | 46.29 | C38H32O15 | P | 729.18139 | 729.17950 | -2.601 | 567.13, 135.04 | Flavonoids |
| 75 | Ginkgetin-7-O-Glc isomer | 46.96 | C38H32O15 | P | 729.18139 | 729.17920 | -3.012 | 567.13, 135.04 | Flavonoids |
| 76 | Genkwanin^*^ | 46.99 | C16H12O5 | N | 283.06119 | 283.06116 | -0.130 | 268.04, 240.04, 211.04, 151.00, 117.03 | Flavonoids |
| 77 | Bilobetin isomer | 48.11 | C31H20O10 | P | 553.11292 | 553.11200 | -1.669 | 435.07, 403.04, 391.08, 297.07, 153.02, 121.03 | Flavonoids |
| 78 | Ginkgetin | 48.78 | C32H22O10 | P | 567.12857 | 567.12762 | -1.681 | 417.06, 167.03, 121.03 | Flavonoids |
| 79 | Isoginkgetin | 48.94 | C32H22O10 | P | 567.12857 | 567.12756 | -1.787 | 417.06, 167.03, 121.03 | Flavonoids |
| 80 | Sciadopitysin | 50.19 | C33H24O10 | P | 581.14422 | 581.14294 | -2.208 | 449.08, 417.06, 167.03, 121.03 | Flavonoids |
| 81 | 2,3-Dihydrosciadopitysin | 50.23 | C33H26O10 | P | 583.15987 | 583.15826 | -2.767 | 417.13, 167.03, 135.04 | Flavonoids |

^*^compounds were identified by reference standards; P, positive ion mode; N, negative ion mode.
