# Supplementary figures and images for: Comprehensive metabolomics and lipidomics profiling uncovering neuroprotective effects of Ginkgo biloba L. leaf extract on Alzheimer’s disease
Source: Front Pharmacol. 2022 Dec 21;13:1076960. doi: 10.3389/fphar.2022.1076960 (PMC9810818; doi:10.3389/fphar.2022.1076960)

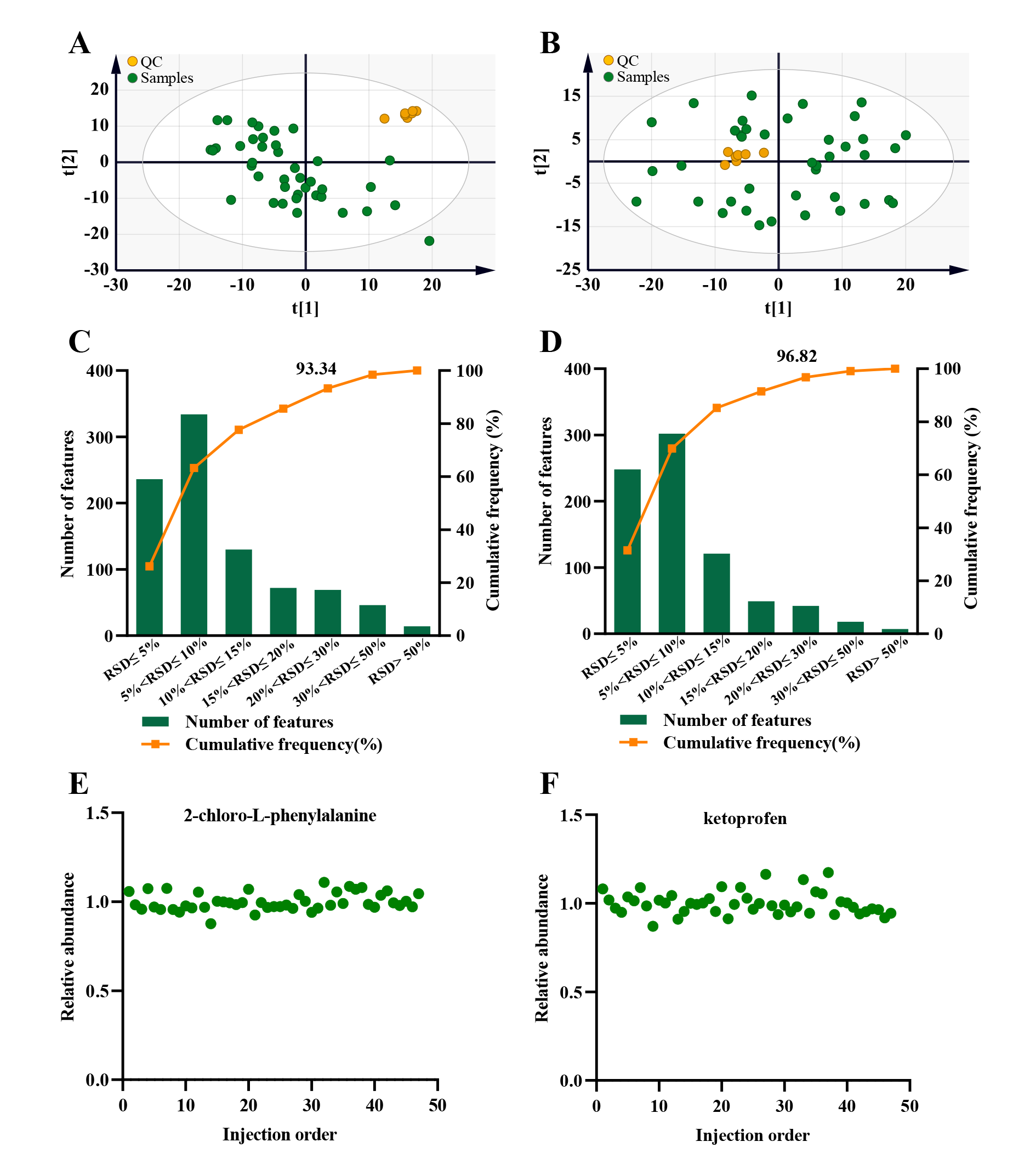

Supplement: Supplementary file 2 [file Image3.TIF]

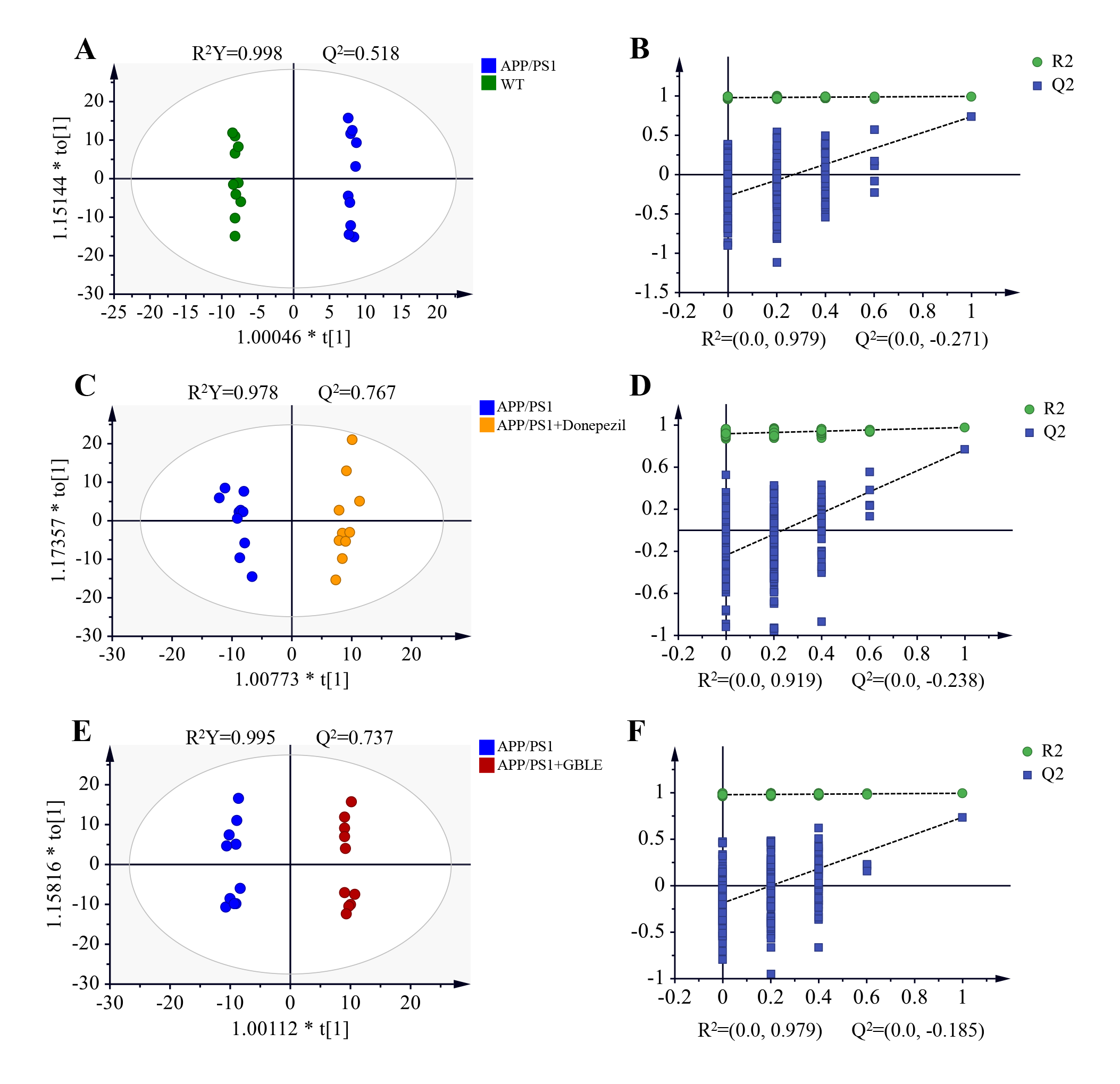

Supplement: Supplementary file 3 [file Image4.TIF]

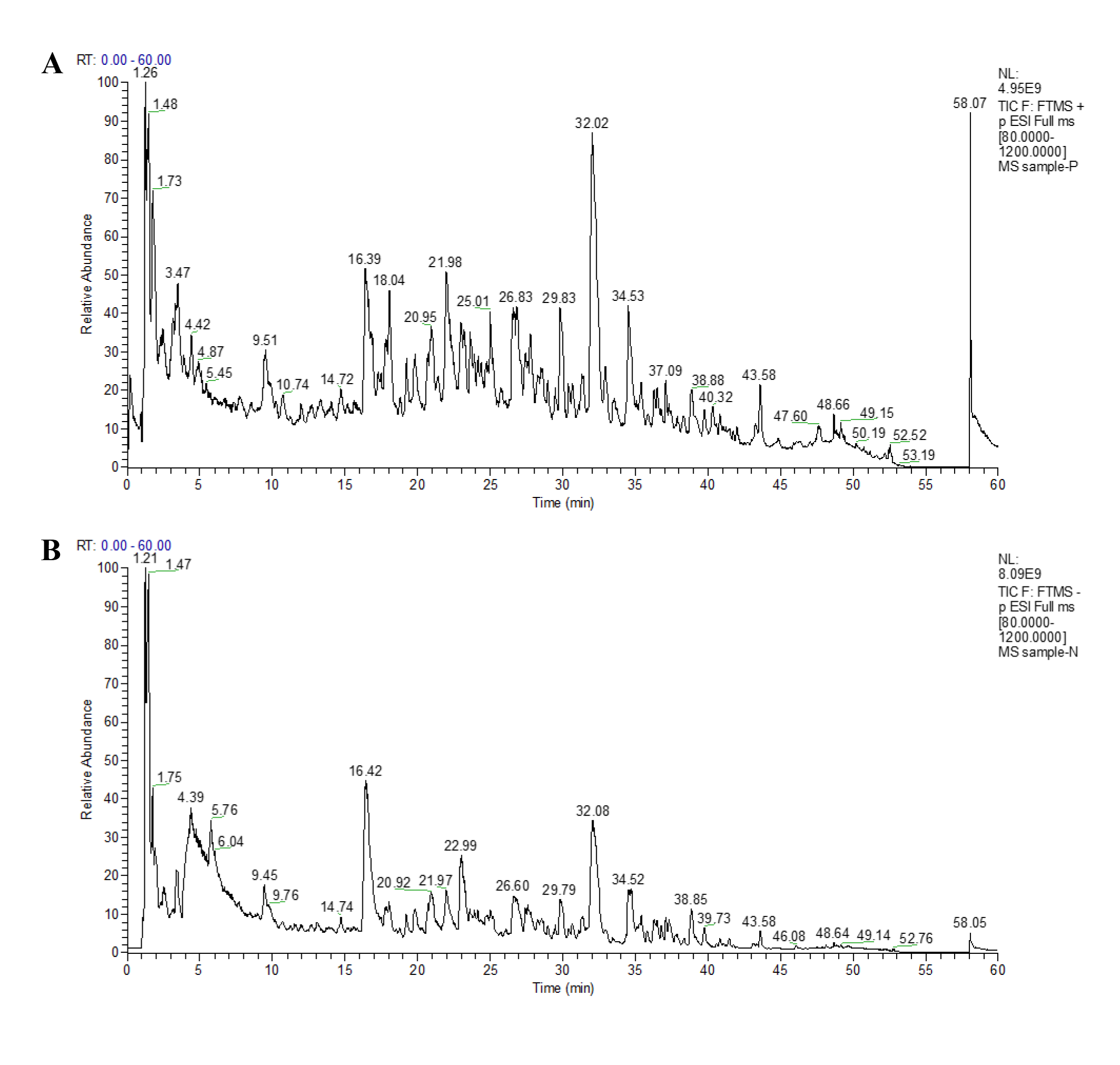

Supplement: Supplementary file 4 [file Image2.TIF]

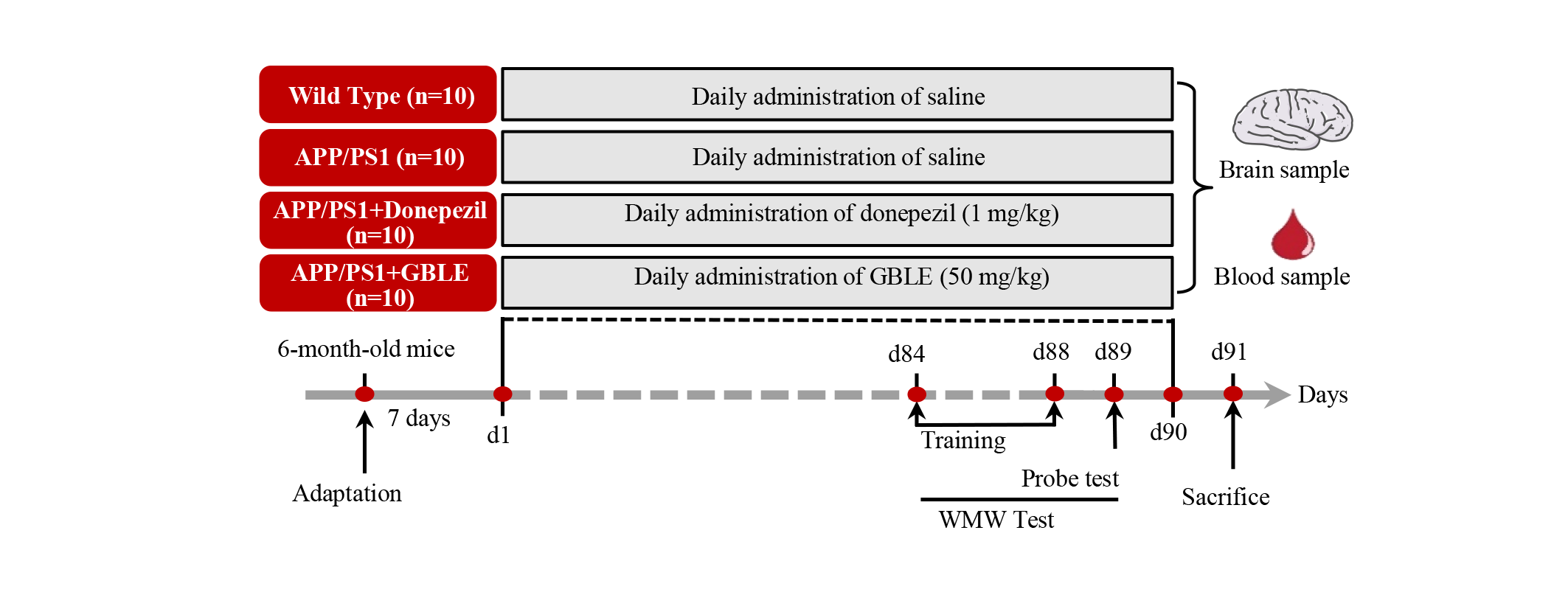

Supplement: Supplementary file 5 [file Image1.TIF]
